# Insights Into CO_2_ Loss, pH Effects, and Tafel Kinetics in Ni Single Atom‐Driven Bicarbonate Electroreduction

**DOI:** 10.1002/advs.202524353

**Published:** 2026-02-08

**Authors:** Lin Li, Yi‐Jie Kong, Ting Zhang, Xu Han, Kristine Aalestrup, Steen Uttrup Pedersen, Xin‐Ming Hu, Kim Daasbjerg

**Affiliations:** ^1^ Novo Nordisk Foundation CO_2_ Research Center Department of Chemistry Aarhus University Aarhus Denmark; ^2^ Environment Research Institute Shandong University Qingdao China

**Keywords:** bicarbonate conversion, CO_2_ loss, pH effects, kinetics, Tafel analysis

## Abstract

Electrochemical reduction of bicarbonate offers an attractive pathway for converting captured CO_2_ into valuable chemicals under mild conditions, thereby bypassing prior CO_2_ release. Herein, we examine the catalytic performance and mechanism of a Ni single‐atom catalyst in bicarbonate electrolysis to CO and H_2_, emphasizing the impact of CO_2_ (carbon species) escaping from the electrolyte, a critical and often‐overlooked factor in the literature that affects selectivity and efficiency. We investigate three cell configurations: (1) closed cell with no CO_2_ escape, preserving reactive carbon species; (2) open cell with moderate escape, causing gradual depletion; and (3) Ar‐purged cell with significant escape, accelerating degassing and losses. In an H‐cell, however, CO selectivity declines over time in both open and Ar‐purged setups due to changes in the electrolyte. Infrared spectroscopy, pH monitoring, and quantitative carbonate‐speciation analysis indicate that loss of CO selectivity stems from the depletion of reactive carbon species (dissolved CO_2_ from bicarbonate dissociation) and buffer shifts, rather than catalyst deactivation. Selectivity is restored by pH adjustment. Kinetic analyses, including Tafel slopes (∼118 mV dec^−1^) and electrochemical impedance spectroscopy, reveal a rate‐determining step in which a pre‐equilibrium chemical reaction is coupled to electron transfer to adsorbed CO_2_ intermediates.

## Graphical abstract

Selective CO formation in bicarbonate electrolysis is achieved by retaining reactive CO_2_ and tuning pH, while the competing hydrogen evolution reaction occurs at the interface. The rate‐determining step couples the chemical equilibrium of bicarbonate/CO_2_ with electrochemical charge transfer, offering insight into optimizing reaction conditions for enhanced bicarbonate conversion.

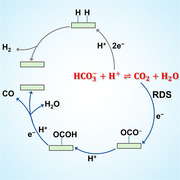

## 1 Introduction

**SCHEME 1:**
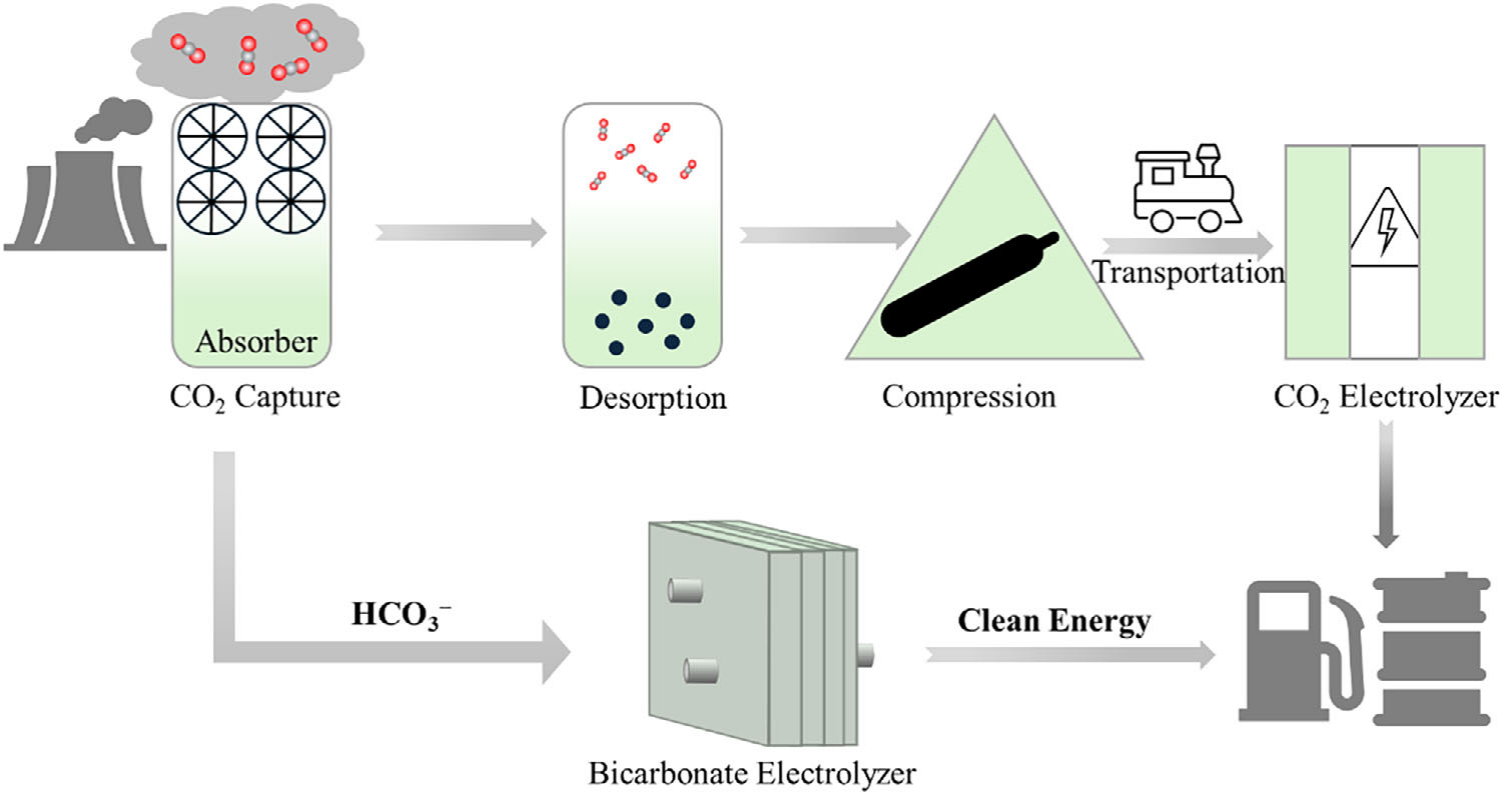
Schematic Representation of the CO_2_ Electrolyzer (Top) and Bicarbonate Electrolyzer (Bottom).

The electrochemical carbon dioxide reduction reaction (eCO_2_RR) represents a sustainable approach to mitigating carbon emissions and storing renewable energy by converting waste CO_2_ into value‐added products [1, 2, 3]. The conventional eCO_2_RR systems typically rely on high‐purity gaseous CO_2_ as feedstock, which necessitates energy‐intensive processes such as gas separation, compression, and transportation (Scheme 1) [4, 5]. To avoid additional energy consumption, attention has been directed toward integrated carbon capture and utilization strategies, particularly the direct electrochemical conversion of captured carbon [6, 7, 8]. Bicarbonate (HCO_3_^−^), as a stable and soluble form of CO_2_ capturing in alkaline media, can be utilized as an alternative precursor for electroreduction [9, 10]. Electrochemical bicarbonate conversion enables direct integration with carbon capture systems without prior CO_2_ release and operation under ambient conditions, potentially lowering the energy and economic barrier associated with CO_2_ utilization (Scheme 1) [6, 11].

Based on existing studies, electrochemical bicarbonate conversion is fundamentally governed by the equilibrium between HCO_3_^−^ and dissolved CO_2_, with CO_2_ serving as the actual electroactive species [11, 12, 13]. Consequently, bicarbonate‐fed electrolysis relies on the CO_2_ generation from bicarbonate. This feature underscores the importance of understanding the factors governing CO_2_ availability and reactivity, which are critical to bicarbonate conversion. Numerous catalysts have been explored for the eCO_2_RR, many of which exhibit high activity and selectivity toward specific products [14, 15, 16]. Among them, Ni single‐atom catalysts (SACs) excel in CO_2_‐to‐CO conversion under gaseous CO_2_ feed, achieving Faradaic efficiencies (FEs) exceeding 90 % under optimized conditions [16, 17, 18, 19, 20].

However, bicarbonate feedstocks yield poorer stability and lower performance than gaseous systems [21, 22], primarily due to the complex equilibria among dissolved inorganic carbon (DIC) species (e.g., HCO_3_^−^ and CO_3_^2−^), governed by pH, potential, and gaseous environment [23, 24]. Although the electrolyzer design [25] and electrode modification [26] have improved bicarbonate electrolysis, fundamental insights into electrolyte effects on reaction pathways and product distribution remain limited. Such dynamic conditions also obscure the rate‐determining step (RDS), impeding rational catalyst/electrolyzer optimization. Elucidating the intrinsic mechanism is thus essential for developing efficient, stable bicarbonate conversion [27, 28, 29].

Herein, we investigate the mechanism of bicarbonate electrolysis at a Ni–SAC cathode. This Ni SAC features atomically dispersed Ni sites anchored on hollow carbon spheres, a distinctive architecture that, as demonstrated in our previous work, enables excellent performance for eCO_2_RR, achieving an FE_CO_ of 98 % at −0.97 V vs. RHE in an H‐cell [30]. On this basis, we extend the application of the same Ni SAC to electrochemical bicarbonate conversion, systematically evaluating its electrochemical performance and identifying the RDS. Three cell configurations are considered: (1) closed cell with no CO_2_ escape, preserving reactive carbon species, (2) open cell with moderate escape, causing gradual depletion, and (3) Ar‐purged cell with significant escape, accelerating degassing and losses. In an Ar‐purged H‐cell, FE_CO_ exceeds 60 % (with H_2_ as the other product), indicating high activity, but the CO selectivity declines significantly over time due to changes in the electrolyte composition. Infrared (IR) spectroscopy indicates that dissolved CO_2_ from equilibrium processes is the active reactant, with electrolysis degradation stemming from CO_2_ depletion and alkalization. This effect is often mitigated in bicarbonate‐fed eCO_2_RR studies by periodic electrolyte refreshment [26, 31]; a careful quantification of the pH effect reveals that it can likewise be done by pH restoration. Tafel analysis and electrochemical impedance spectroscopy (EIS) indicate a RDS involving chemical pre‐equilibrium coupled with electron transfer. These findings clarify the role of electrolyte composition in bicarbonate electrolysis and guide stability enhancements through electrolyte management and catalyst design.

## 2 Results and Discussion

### 2.1 Effects of Gas Purging on Electrolyte

In most bicarbonate electrolysis, the system is continuously purged with an inert gas (e.g., Ar), but the effect of gas exchange is rarely examined. Therefore, our primary focus was on three configurations:

#### 2.1.1 Closed Cell

**FIGURE 1:**
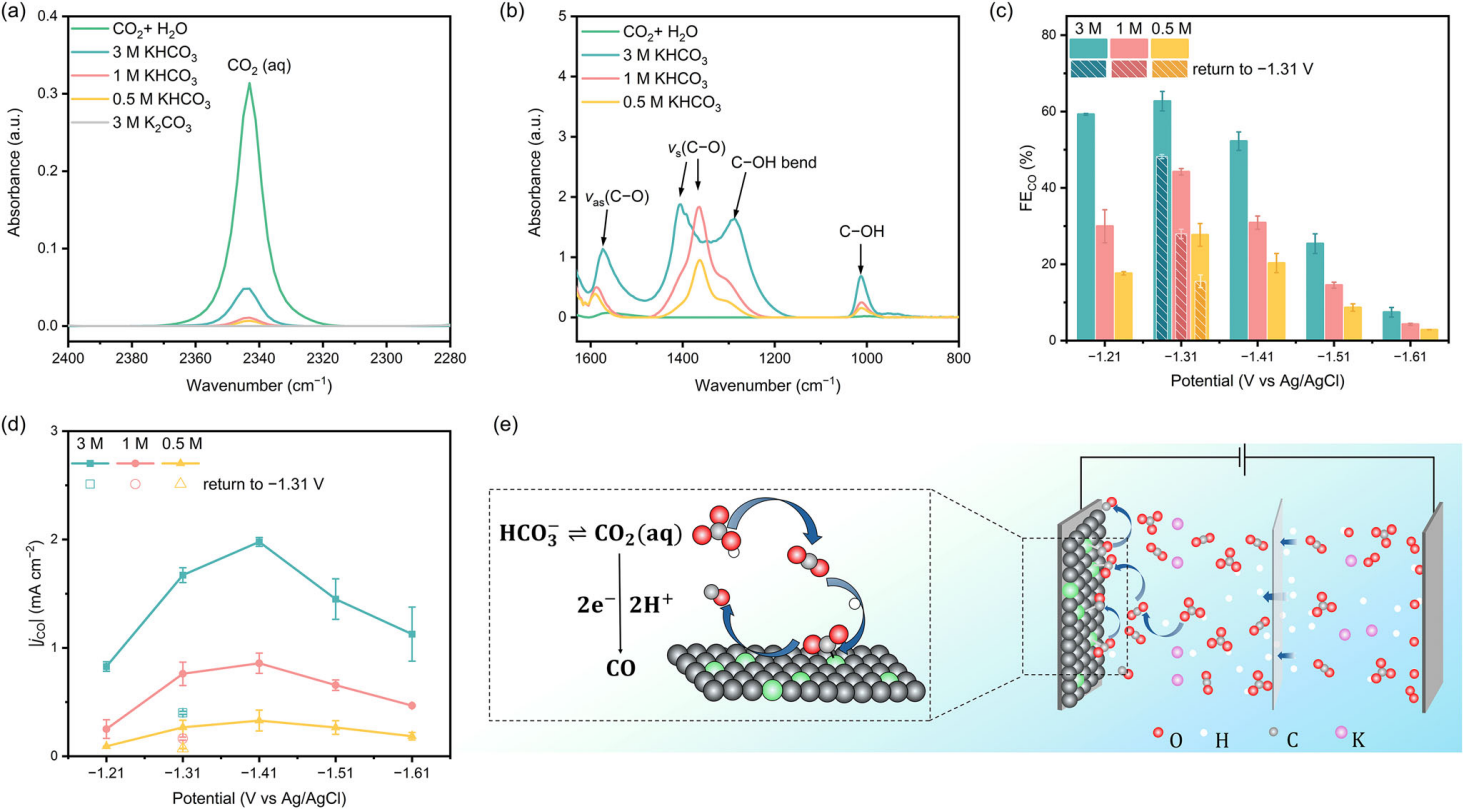
IR spectra of Ar‐saturated KHCO_3_ solutions (after 30 min of Ar purging at 30 mL min^−1^) of varying concentrations, highlighting signals for (a) CO_2_(aq), and (b) bicarbonate/carbonate species corresponding to the closed‐cell configuration. (c) FE_CO_ and (d) |*j*_CO_| recorded at progressively negative potentials at varying electrolyte concentration and potential in an open H‐cell configuration, with the electrolyte purged with Ar (30 mL min^−1^) for 30 min before commencing electrolysis; the plotted values correspond to the arithmetic mean of measurements collected at 10 and 27.5 min of electrolysis; slashed bars and discrete points represent the results measured after returning to −1.31 V vs. Ag/AgCl at the end. (e) Schematic of the electrochemical bicarbonate conversion.

IR spectroscopy was used to quantify the dissolved CO_2_ concentration in KHCO_3_ solutions of varying concentrations, with Ar‐saturated ultrapure water as the background and CO_2_‐saturated ultrapure water as the reference. To elucidate the effect of purging, recordings were performed 30 min after Ar purging (flow rate = 30 mL min^−1^). IR measurements were taken immediately after the solution was injected into the closed cell. The infrared absorption peak at 2343 cm^−1^, attributed to C═O stretching in CO_2_(aq) [32, 33], appears in all solutions except Ar‐saturated 3 m K_2_CO_3_ (Figure 1a). As shown, the absorbance peak of CO_2_(aq) in bicarbonate solutions increases with increasing bicarbonate concentration but remains significantly lower than that in CO_2_‐saturated water.

Likewise, the IR signals at lower wavenumbers (Figure 1b) depend on the KHCO_3_ concentration. At low concentrations, a prominent absorption band is observed at 1365 cm^−1^ corresponding to the symmetric CO stretching vibrations, with a minor shoulder at 1297 cm^−1^ attributable to the COH bending [34, 35, 36]. At 3 m, distinct bands appear at 1406 and 1288 cm^−1^. In contrast, the 3 m K_2_CO_3_ spectrum shows the strongest absorption at 1327 cm^−1^ (Figure S1a), consistent with asymmetric C─O bond stretching of free CO_3_^2−^ [34, 37]. The absence of an absorption near 1290 cm^−1^ in K_2_CO_3_ indicates that the 1288 cm^−1^ peak in 3 m KHCO_3_ arises from HCO_3_^−^ under solvation and ion‐pairing conditions (COH bending), not CO_3_^2^^−^ from equilibrium. A peak at 1575 cm^−1^, from asymmetric CO vibration of HCO_3_^−^ with partial C═O character, is shifted relative to low‐concentration spectra. In concentrated electrolytes, ion association and hydrogen bonding shift vibrational modes in anions (e.g., NO_3_^−^, HCOO^−^), as confirmed by ATR‑FTIR and 2D‑IR studies [37, 38]. A characteristic peak at 1010 cm^−1^ in all bicarbonate solutions is assigned to C─OH stretching in HCO_3_^−^ [39, 40].

Based on the known concentration of CO_2_ in CO_2_‐saturated water (33 mm) as a reference, with CO_2_(aq) quantified from the peak area (Figure S1b), the CO_2_(aq) concentration can be extracted for any of the solutions after 30 min of Ar purging (Table S1) [41, 42]. As seen, the CO_2_(aq) concentration in 3 m KHCO_3_ is ∼9 times (i.e., 4.88 mm) higher than that in 0.5 m KHCO_3_ (i.e., 0.51 mm), though both are significantly below 33 mm because of the effect from the prior 30 min purging with Ar. The spectral profiles obtained after an additional 30 min are identical, i.e., no changes are detected in any of the solutions with time in a closed IR liquid cell, including the CO_2_(aq) absorption (Figure S1c–f) or signals from other DIC species (Figure S2), due to a rapid establishment of equilibrium between bicarbonate and CO_2_(aq) within 1−2 min.

#### 2.1.2 Open Cell

In the open‐cell configuration, the 3 m KHCO_3_ solution is exposed to the atmosphere without gas purging, and pH was monitored over time under non‐electrolytic conditions. The measured pH, together with the assumption of constant total alkalinity, was used to calculate DIC concentrations [43, 44]. In contrast to the time‐independence of the CO_2_(aq) concentration observed in the closed‐cell setup, the pH changes significantly from 8.30 initially to 8.65 after 4 h (Figure S3), with [CO_2_(aq)] progressively decreasing from 0.033 m (equal to that of CO_2_‐saturated pure water) to 0.014 m (Table S2). At the same time, [DIC] declines from 3.000 to 2.949 m, corresponding to a < 2 % carbon loss to the atmosphere.

#### 2.1.3 Ar‐Purged Open Cell

To assess the effect of gas purging on carbon loss, the open‐cell system was purged with Ar continuously (flow rate = 30 mL min^−1^). As shown in Table S3, a pronounced increase in pH is observed within the first hour in the KHCO_3_ solution, indicating a substantially higher alkalization rate at the early stage. After 4 h, the carbon loss to the atmosphere accelerates, i.e., [CO_2_(aq)] progressively decreases from 0.033 to 0.004 m (Table S3), while [DIC] declines from 3.000 to 2.813 m, corresponding to a loss of 0.187 m carbon species. Bicarbonate (HCO_3_^−^) remains dominant, but decreases gradually as the pH rises, while CO_3_^2−^ increases. Compared with the ∼2 % carbon loss in the open‐cell setup, Ar‐purged conditions result in ∼7 % carbon loss via CO_2_ degassing over 4 h (Figure S4). More rapid carbon loss is expected at higher Ar flow rates, due to accelerated CO_2_ degassing under stronger purge conditions.

### 2.2 Performance of Bicarbonate‐Fed eCO_2_RR

IR spectroscopy revealed that higher KHCO_3_ concentrations increase the equilibrium concentration of dissolved CO_2_, which is expected to increase the CO production rate under otherwise identical electrochemical conditions. This effect would be most pronounced in a closed‐cell configuration; however, gas evolution and the resulting pressure buildup render such a setup impractical, particularly when in‐line gas chromatography is required for product quantification. Consequently, electrochemical performance was evaluated in an Ar‐purged cell to quantify the overall effects of carbon loss from both purging and the eCO_2_RR process.

We first evaluated 0.5, 1, and 3 m KHCO_3_ electrolytes over a potential range from −1.61 to −1.21 V vs. Ag/AgCl in an H‐cell (Figure 1c,d), with the electrolyte purged with Ar for 30 min before commencing electrolysis. Electrolysis yields CO and H_2_ via the competing hydrogen evolution reaction (HER), but in this study, we focus exclusively on CO. As shown, the electrochemical performance improves markedly with increasing KHCO_3_ concentration, i.e., the 3 and 1 m KHCO_3_ systems yield significantly higher FE_CO_ and absolute CO partial current density, |*j*_CO_|, than the 0.5 m electrolyte. The low CO selectivity in 0.5 m KHCO_3_ (Figure S5) and the negligible CO formation and current in 3 m K_2_CO_3_ (Figure S6) suggest that neither bicarbonate nor carbonate serves as an electroactive species. Taken together with the IR data, these results indicate that CO_2_ generated from the bicarbonate equilibrium acts as the actual reactive species (Figure 1e).

In 3 m KHCO_3_, FE_CO_ exceeds 60 % at −1.31 V vs. Ag/AgCl (−0.603 V vs. RHE). This enhancement arises from increased bicarbonate decomposition, which elevates the concentration of reactive CO_2_(aq) near the electrode surface and promotes faster CO_2_ reduction. Regarding the performance of our Ni‐SAC for bicarbonate conversion, the FE_CO_ of 63 % under optimal conditions is comparable to values reported for MOF‐derived Ni SAC (67 %) [21], Ni tetraphenylporphyrin immobilized on carbon black (70 %) [45], Co phthalocyanine on carbon nanotube (85 %) [25], and classical Ag nanoparticles catalyst (37 %) [10] under an open flow‐cell configuration. The maintained selectivity demonstrates that our Ni SAC has sufficient potential for bicarbonate conversion. The FE_CO_ and |*j*_CO_| display volcano‐type potential dependence (Figure 1c,d; Figure S7). At low overpotentials, the CO_2_ reduction is kinetically limited. More negative potentials enhance adsorption/activation of CO_2_‐derived intermediates on the Ni SAC, promoting CO formation. However, at excessively negative potentials, the limited availability of CO_2_(aq) favors HER, reducing both selectivity and the reaction rate for CO production. As a result, the absolute overpotential for maximum CO production is smaller than for the conventional gaseous CO_2_ electroreduction (−0.97 V vs. RHE) [30].

**FIGURE 2:**
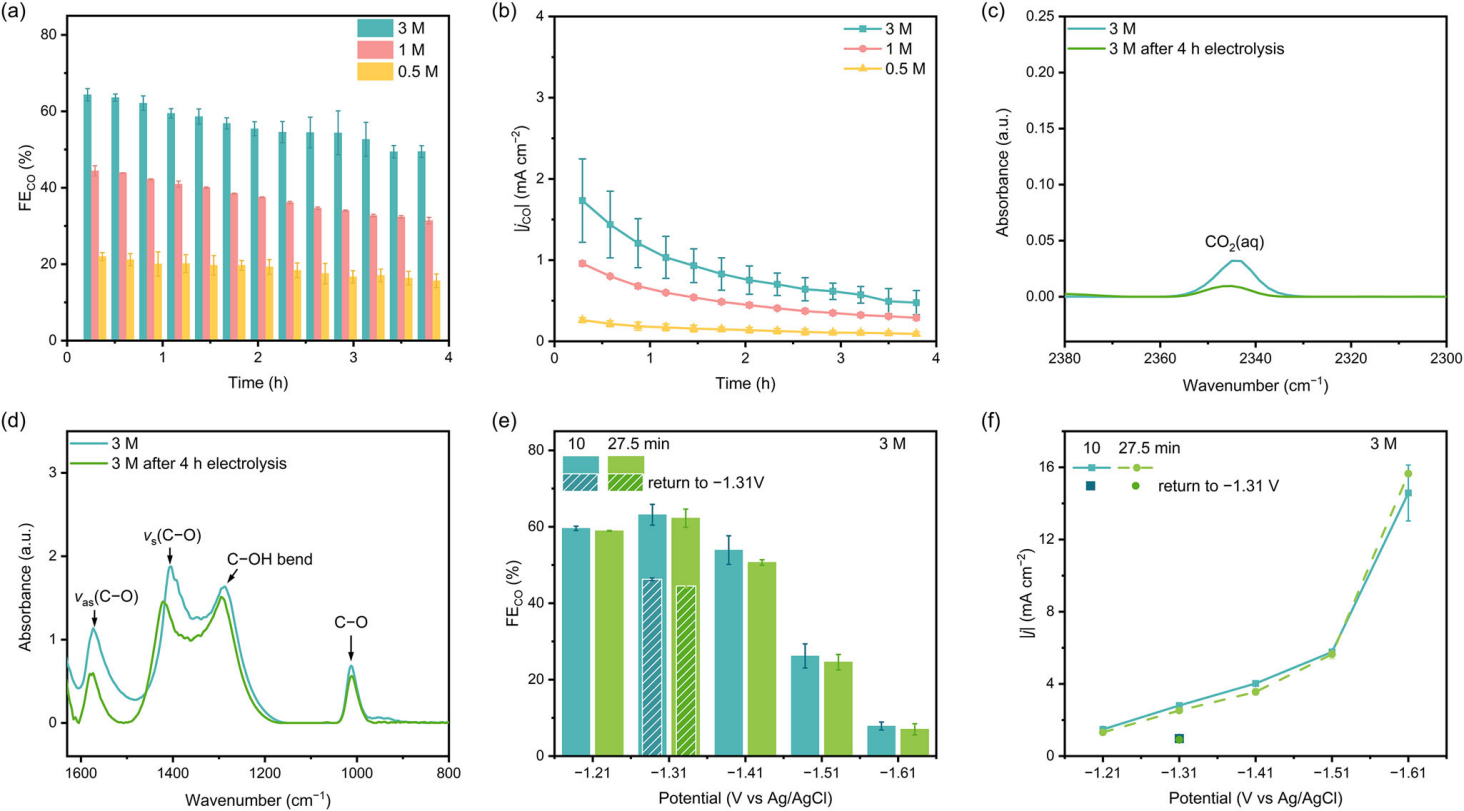
(a) FE_CO_ and (b) |*j*_CO_| during 4 h electrolysis at −1.31 V vs. Ag/AgCl in KHCO_3_ solutions of varying concentrations in an Ar‐purged H‐cell [i.e., the open‐cell configuration with the electrolyte purged with Ar (30 mL min^−1^) for 30 min before commencing electrolysis]. IR signal of (c) dissolved CO_2_(aq) and (d) bicarbonate and carbonate species before and after 4 h electrolysis. (e) FE_CO_ and (f) |*j*| measured at 10 min and 27.5 min of electrolysis at progressively negative potentials in an Ar‐purged H‐cell with 3 m KHCO_3_; slashed bars in (e) and discrete points (closely spaced) in (f) represent the results measured upon returning to −1.31 V vs. Ag/AgCl at the end.

After performing electrolysis at −1.61 V vs. Ag/AgCl in 3 m KHCO_3_, the potential was reset to −1.31 V vs. Ag/AgCl (slashed bars in Figure 1c). This results in poorer electrochemical performance than that of the initial measurement at −1.31 V, with FE_CO_ decreasing from 63 % to 42 % and |*j*_CO_| declining from 1.67 to 0.40 mA cm^−2^ (discrete points in Figure 1d) because of decreased operational stability. To further evaluate the performance stability of Ni SAC in bicarbonate conversion, electrolysis experiments were conducted at −1.31 V vs. Ag/AgCl for 4 h in Ar‐saturated KHCO_3_ electrolyte, with product analysis every 17.5 min. As shown in Figure 2a,b, both FE_CO_ and |*j*_CO_| increase with bicarbonate concentration at all time points. In 3 m KHCO_3_, FE_CO_ remains above ∼50 % throughout the electrolysis period, yielding syngas with a CO: H_2_ ratio of 1:1 [10, 46]. Meanwhile, FE_CO_ declines to 31 % and 16 % at the end of the 4 h electrolysis in the 1 m and 0.5 m KHCO_3_, respectively, with a decrease of |*j*| (Figure S8).

Earlier studies have demonstrated that other electrolyte components, such as K^+^, can significantly influence the performance [47, 48]. To investigate the effects of K^+^, we employed a KHCO_3_ electrolyte of varying molarities, and KCl was supplemented to maintain a total [K^+^] of 3 m. As shown in Figure S9a,b, mixed KHCO_3_−KCl electrolytes yield no improvement in FE_CO_ over pure KHCO_3_, despite higher |*j*| from increased ionic strength (Figure S9c). In contrast, |*j*_CO_| is slightly lower in KHCO_3_−KCl mixtures than in pure 1 and 0.5 m pure KHCO_3_ (Figure S9d), resulting in reduced FE_CO_ across potentials (Figure S9e). These findings suggest that [K^+^] does not determine the CO selectivity or activity under the tested conditions. Although previous studies have reported that K^+^ can modulate the interfacial electric field and stabilize intermediates in conventional CO_2_‐saturated systems [49, 50, 51], its effect is limited in our setup, where the CO_2_(aq) availability governed by the bicarbonate equilibrium dominates. Consistent with previous multi‐potential measurements, control experiments in which the equalized KHCO_3_/KCl electrolyte combinations with respect to K^+^ show negligible differences (Figure S10), confirming that the gradual decrease of electrochemical performance is intrinsic to the system rather than caused by variations in [K^+^].

Post‐electrolysis FTIR spectra of the 3 m KHCO_3_ electrolyte reveal diminished absorbance at 2343 cm^−1^ (CO_2_(aq) asymmetric stretching, Figure 2c), indicating depletion of dissolved CO_2_. The decreased intensity of the peak at 1010 cm^−1^ (C─OH stretching vibration of HCO_3_^−^, Figure 2d) suggests the lower concentration of HCO_3_^−^ after electrolysis. Similar trends occur in lower‐concentration electrolytes (Figure S11). These observations confirm that prolonged electrolysis alters the electrolyte composition, thereby limiting the availability of CO_2_(aq) and affecting the performance of bicarbonate conversion.

To quantify the contribution of electrolysis to carbon loss, we compared pH changes under Ar purging alone vs. during electrolysis. In both cases, spontaneous liberation of CO_2_(g) occurs due to Ar purging, and the process is largely similar whether electrolysis is applied or not. With electrolysis, the pH rises from 8.30 to 9.19 after 2 h, an increase of 0.15 units relative to the non‐electrolyzed condition. This enhanced rise is caused by proton consumption at the cathode during the reduction of CO_2_ to CO and the generation of H_2_, which removes protons from the solution and thereby shifts the equilibrium. Electrochemical conversion accelerates alkalinization of the electrolyte, thereby limiting the availability of CO_2_(aq) at an earlier stage and consequently constraining long‐term operational stability.

These results confirm that the spontaneous pH increase, driven primarily by CO_2_ removal, markedly reduces CO_2_(aq) availability and is the leading cause of the performance degradation during prolonged electrolysis. For instance, disregarding electrolysis, the [CO_2_(aq)] declines from 0.011 m (after 30 min of Ar purging, corresponding to the start of electrolysis if it were applied) to 0.006 m (2 h purging), and further to 0.004 m (4 h purging; Table S3), leaving little CO_2_(aq) for electroreduction at longer purging times. This purging effect is even reflected in the FE_CO_ values reported in Figure 1c, which are the averages of measurements taken at 10 and 27.5 min of electrolysis. In fact, FE_CO_ decreases consistently from 10 to 27.5 min at all studied potentials (Figure 2e), with the effect being more pronounced at larger overpotentials. The absolute current densities show little difference between 10 and 27.5 min, as HER contributes significantly (Figure 2f). These results further demonstrate that electrochemical performance is rapidly affected by the evolving electrolyte conditions.

### 2.3 Restoration of Bicarbonate‐Fed eCO_2_RR

#### 2.3.1 pH Restoration

**FIGURE 3:**
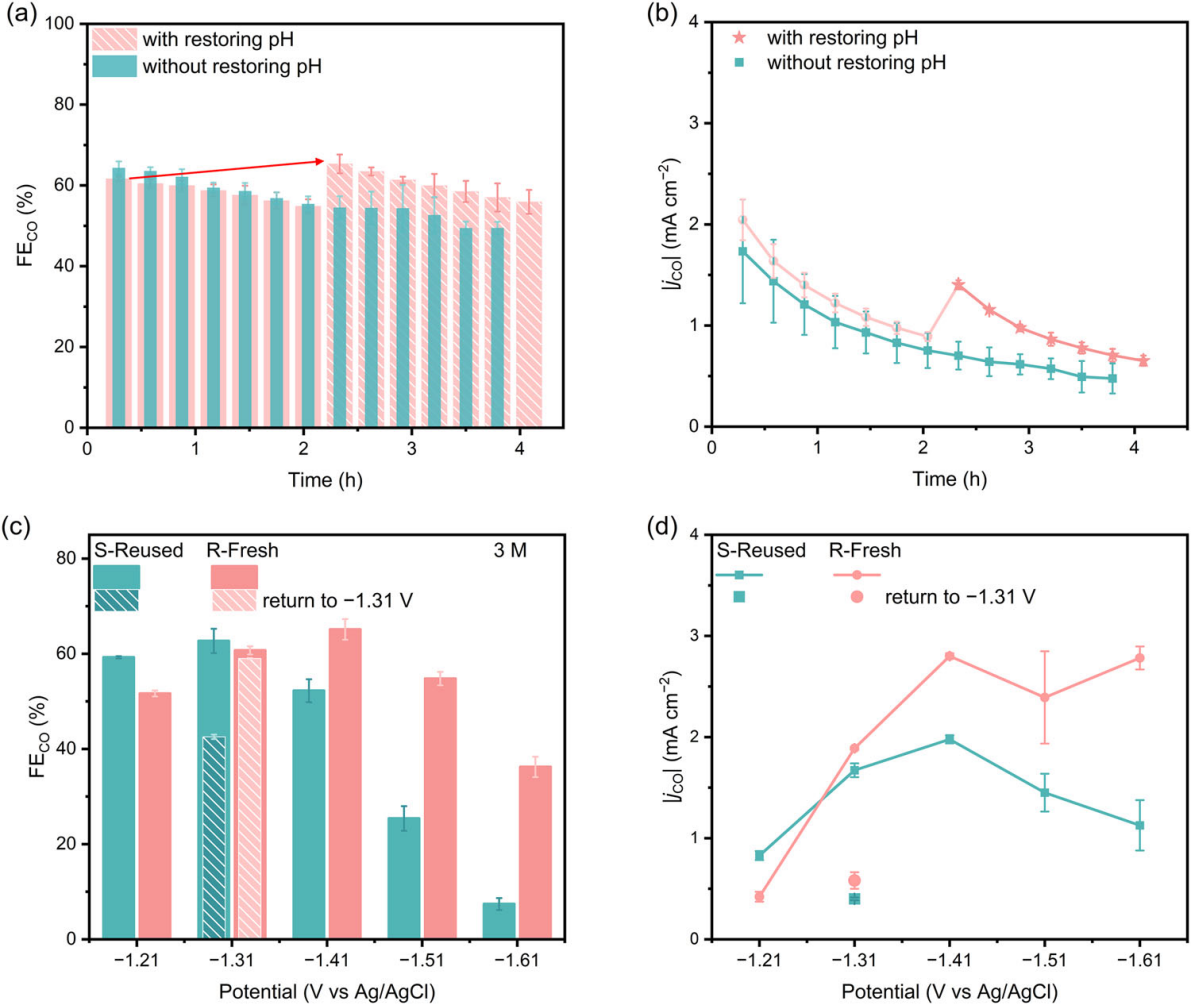
(a) FE_CO_ and (b) |*j*_CO_| with and without pH adjustment during 4 h electrolysis at −1.31 V vs. Ag/AgCl in 3 m KHCO_3_. (c) FE_CO_ and (d) |*j*_CO_| obtained from controlled‐potential electrolysis at various potentials using a Ni SAC catalyst in Ar‐saturated 3 m KHCO_3_; the slashed bars in (c) and discrete points in (d) represent the results measured upon ultimately returning to −1.31 V vs. Ag/AgCl. Two experimental protocols were compared: R‐Fresh, in which a randomized potential sequence (−1.31, −1.21, −1.41, −1.61, −1.51, and −1.31) and fresh electrolyte were used for each run; S‐Reused, involving a sequentially decreasing potential order (−1.21, −1.31, −1.41, −1.51, −1.61, and −1.31) in an unreplaced electrolyte batch. In both protocols, the same working electrode was used throughout all potential steps, and each potential was held for 30 min.

Since pH governs the acid‐base equilibrium between bicarbonate, carbonate, and CO_2_, such changes can be exploited to influence the availability of CO_2_(aq) at the catalyst‐electrolyte interface and subsequent reduction [52, 53]. To verify this, bulk electrolyte pH was restored during electrolysis at −1.31 V vs. Ag/AgCl. pH increases from 8.36 to 9.19 with the first 2 h of electrolysis, accompanied by a gradual decline in FE_CO_ (Figure 3a, pink bars). After 2 h, the pH was restored to its initial value by adding an appropriate amount of 1 m HCl, and electrolysis continued for an additional 2 h under identical conditions (Figure S12). FE_CO_ recovered to 67 % (Figure 3a, pink slashed bars), comparable to the initial value (62 %), while FE_CO_ without pH adjustment continued decreasing (Figure 3a, blue bars). A slight initial improvement in |*j*_CO_| is also observed (Figure 3b, pink stars). These results demonstrate that declining CO selectivity primarily stems from rising pH, which shifts the equilibrium among the DIC species toward CO_3_^2−^ and depletes CO_2_(aq). pH restoration reverses this effect, underscoring the critical role of pH in sustaining the availability of CO_2_(aq) and CO formation.

#### 2.3.2 Electrolyte Refreshment

Periodic replacement of the KHCO_3_ solution has been shown previously to sustain the efficiency of eCO_2_RR [26, 31]. Figure 3c shows that electrolysis (S‐Reused) with the same electrolyte at sequentially negative potentials from −1.21 to −1.61 V vs. Ag/AgCl yields FE_CO_ <10 % at −1.61 V vs. Ag/AgCl, with no recovery to initial values upon returning to −1.31 V vs. Ag/AgCl. In contrast, use of fresh 3 m KHCO_3_ for each step (R‐Fresh) maintains high FE_CO_ throughout. At low overpotentials, performance differences between R‐Fresh and S‐Reused protocols are negligible. However, at more negative potentials, R‐Fresh shows marked improvement. For example, at −1.61 V vs. Ag/AgCl, FE_CO_ increases to 36 % in R‐Fresh relative to 7 % in S‐Reused. Remarkably, even after stepping from −1.61 V to −1.51 V vs. Ag/AgCl, R‐Fresh achieves FE_CO_ = 55 %. Upon returning to −1.31 V vs. Ag/AgCl with fresh electrolyte (slashed pink bar in Figure 3c), FE_CO_ matches the initial value of 61 % at this potential. At the same time, |*j*_CO_| increases with more negative potentials in both protocols (Figure 3d), peaking at −1.41 V vs. Ag/AgCl. These results confirm that performance degradation arises primarily from changes in electrolyte composition rather than irreversible catalyst deactivation. Under R‐fresh conditions, both FE_CO_ and |*j*_CO_| decrease consistently over a short period, with the decline more pronounced at higher overpotentials (Figure S13). These results indicate that the effect of evolving electrolyte conditions on performance is continuous and unavoidable under the studied conditions.

Collectively, these results demonstrate that pH restoration and electrolyte replenishment mitigate the effects of CO_2_ depletion. The above results underscore the critical role of electrolyte composition and pH in maintaining catalytic stability during bicarbonate electrolysis, despite the observed carbon loss. Systematic management of electrolyte composition and pH (e.g., periodic replenishment, controlled pH adjustment) thus offers a practical strategy for improving the long‐term stability and efficiency in bicarbonate electrolysis. Consequently, strategies such as gas recirculation or the development of fully closed systems could be essential to reduce gas‐induced carbon loss and improve carbon retention in scaled‐up or prolonged operation.

### 2.4 Rate‐Determining Step

To substantiate that the mechanism of the selected Ni‐SAC catalyst indeed follows the pathway involving reduction of CO_2_ formed via HCO_3_^−^ dissociation (vide supra), we conducted a detailed mechanistic investigation using Tafel analysis and electrochemical impedance spectroscopy (EIS). These complementary techniques provide critical insights into the kinetics, RDS, and charge‐transfer processes, enabling a rigorous elucidation of the reaction pathway. Measurements were performed on short timescales under controlled conditions in which no carbon loss to the atmosphere occurred, ensuring that observed behaviors reflect intrinsic electrocatalytic processes rather than extraneous electrolyte depletion effects.

*Tafel analysis* is a standard tool in eCO_2_RR kinetics, which relates overpotential to the logarithm of the current density [54, 55]. Here, it was applied under various conditions to quantify the kinetic response and elucidate the reaction pathway, with its rate‐limiting processes (e.g., electron transfer, chemical steps, mass transport) identified on the Ni SAC. Theoretical Tafel slopes, derived assuming quasi‐equilibrated preceding steps for CO_2_ reduction pathways, are directly applicable, with CO_2_(aq) confirmed as the electroactive reactant in the bicarbonate conversion (Table S4) [29, 56].

**FIGURE 4:**
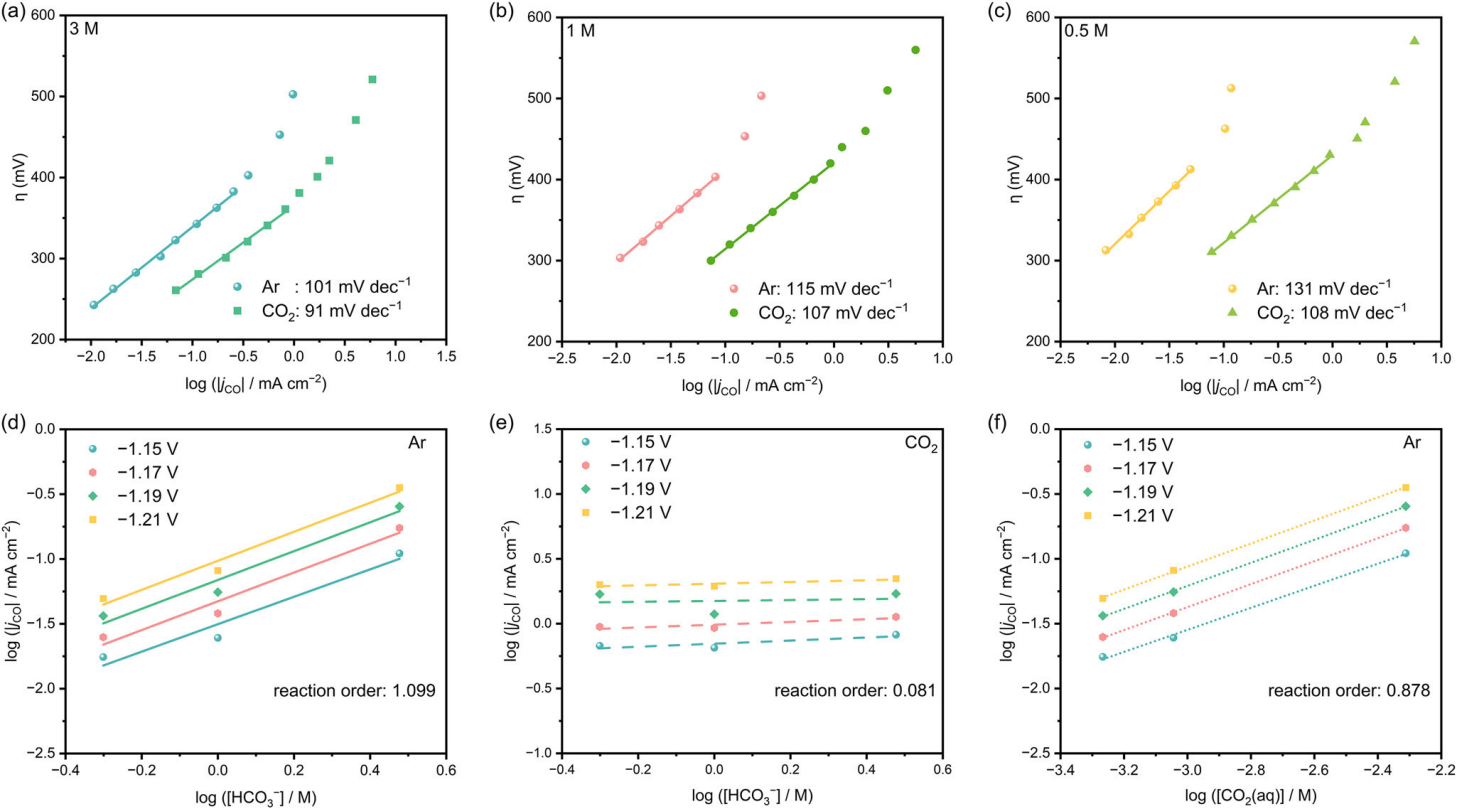
Tafel plots for bicarbonate conversion (Ar) and eCO_2_RR (CO_2_) at a Ni SAC catalyst in KHCO_3_ electrolyte of (a) 3, (b) 1, and (c) 0.5 m. Plot of log |*j*_CO_| vs. log [HCO_3_^−^] at applied potentials of −1.15, −1.17, −1.19 and −1.21 V vs. Ag/AgCl under (d) Ar and (e) CO_2_ atmospheres. (f) Plot of log |*j*_CO_| vs. log [CO_2_(aq)] under Ar atmosphere.

Tafel analysis was performed for both bicarbonate conversion at varying KHCO_3_ concentrations and eCO_2_RR under CO_2_ purging, with measurements conducted at low overpotentials to ensure kinetic control. A constant [K^+^] of 3 m was maintained by adding KCl when needed. Figure 4a–c shows the experimental Tafel plots, with individual fits (overpotential <450 mV) provided in Figures S14 and S15. Under CO_2_‐purged conditions, Tafel slopes range from 91 to 108 mV dec^−1^ across KHCO_3_ concentrations, approaching the theoretical slope 118 mV dec^−1^ for an electron‐transfer (ET) step or, eventually, a proton‐coupled electron‐transfer (PCET) step, depending on the nature of the preceding equilibria. At 3 and 1 m KHCO_3_, the Tafel slopes for bicarbonate conversion and eCO_2_RR are comparable, indicating a steady supply of CO_2_ via equilibria, even under Ar saturation. However, at 0.5 m KHCO_3_, the Tafel slope for bicarbonate conversion increases, rising from 101 to 131 mV·dec^−1^, reflecting slower kinetics due to reduced CO_2_(aq) availability under these conditions. Additionally, the lower buffering capacity results in a substantial increase in pH within the diffusion layer, which further decreases [CO_2_(aq)]. Consequently, the reaction kinetics shift from electrochemical RDS‐limited to CO_2_‐supply‐limited, highlighting the critical role of electrolyte buffer strength in sustaining CO_2_ availability and modulating the kinetics of CO evolution.

Reaction order analysis of |*j*_CO_| with respect to [HCO_3_^−^] was conducted for bicarbonate conversion and eCO_2_RR at −1.15, −1.17, −1.19, and −1.21 V vs. Ag/AgCl (Figure 4d,e). As seen, the plot of log |*j*_CO_| vs. log [HCO_3_^−^] under CO_2_ saturation yields an average slope of 0.081, indicating near‐zero reaction order. This demonstrates that the reaction rate for eCO_2_RR is independent of [HCO_3_^−^], i.e., HCO_3_^−^ is not involved in the RDS. Thus, water is the likely proton source via autoprotolysis or interfacial hydrogen bonding. In addition, the bulk pH dependence under CO_2_ saturation is negligible (slope 0.087 in a plot log |*j*_CO_| vs. pH, Figure S16), which is inconsistent with classical PCET (e.g., B1 or C1 step in Table S4) as the RDS. Taken together, these results indicate that the RDS under a CO_2_ atmosphere is an ET process to an adsorbed CO_2_ intermediate MCO_2_^−^ (eq. 1).

(1) $$\theta_{M}+CO_{2}+e^{-}⇌\theta_{{\mathrm{MCO}}_{2}^{-}}$$

Under Ar‐saturated conditions, |*j*_CO_| showed first‐order dependence on [KHCO_3_] (average slope 1.099) and on [CO_2_(aq)] (Figure 4f). In a bicarbonate system, the chemical pre‐equilibrium between bicarbonate and CO_2_ critically determines the observed kinetics. The first‐order dependence reflects the sensitivity of the CO evolution rate to [HCO_3_^−^] due to the limited equilibrium‐derived CO_2_ availability, which is consistent with larger Tafel slopes (> 118 mV dec^−1^) at lower [KHCO_3_] [57].

Linear fitting of log |*j*_CO_| vs. log [HCO_3_^−^] was performed across all tested potentials (Figure S17). At CO_2_ saturation, the reaction order remains near zero across all potentials (see slopes in Table S5). Under Ar atmosphere, however, the slope increases with more negative potentials (Figure S17d), reflecting a stronger influence of [HCO_3_^−^] on the reaction rate at larger overpotentials. At low overpotentials, slow ET dominates the RDS when reactant supply is sufficient. As the overpotential increases, ET accelerates, and the chemical pre‐equilibrium becomes rate‐limiting. The reaction order in HCO_3_^−^ stays ∼1 in the potential range from −1.15 to −1.21 V vs. Ag/AgCl. The reaction order in HCO_3_^−^ remains ∼1 from −1.21 to −1.15 V vs. Ag/AgCl. The reaction orders support that the pre‐equilibrium step is kinetically involved in the RDS. These observations confirm that a pre‐equilibrium‐coupled electron transfer (PE‐ET) as the RDS for bicarbonate conversion on the Ni SAC, where the RDS involves an ET to adsorbed CO_2_, with CO_2_ supply dictated by the bicarbonate equilibrium.

**FIGURE 5:**
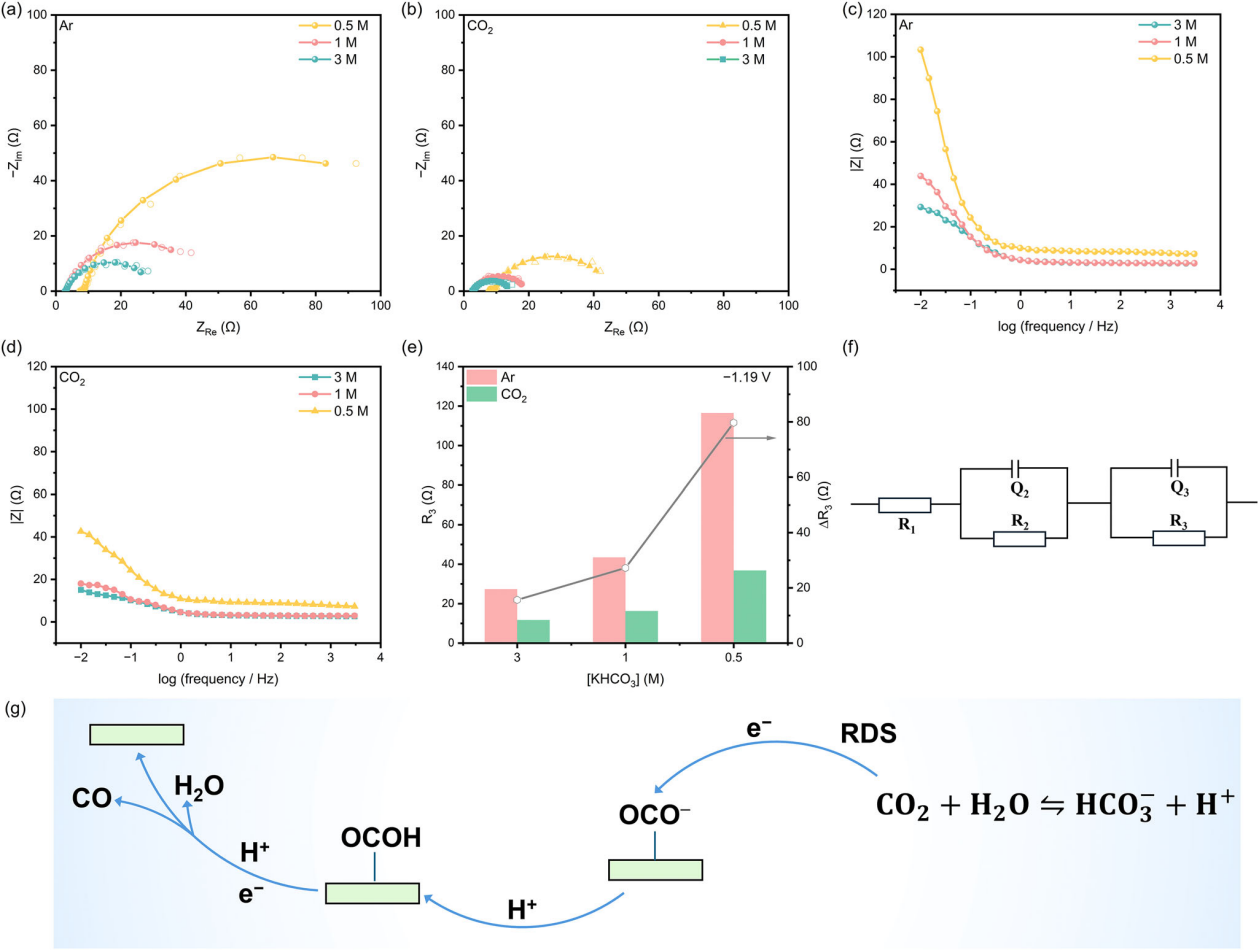
EIS results in Ar‐ and CO_2_‐saturated KHCO_3_ electrolytes of varying concentrations. (a,b) Nyquist plots (symbols) with fitted curves (solid lines) and (c,d) the Bode magnitude under Ar and CO_2_ atmospheres. (e) Extracted *R*_3_ (adsorption‐related charge‐transfer resistance) values under different conditions, with (e) the dependence of the difference in *R*_3_ between CO_2_ and Ar atmospheres on the bicarbonate concentration. (f) The equivalent circuit for fitting: *R*_1_, solution resistance; *R*_2_, high‐frequency interfacial resistance; *R*_3_, the adsorption‐related charge transfer resistance. *Q*_2_, *Q*_3_: constant phase elements, representing non‐ideal capacitive behavior. (g) Schematic the PE–ET RDS. All EIS measurements were performed at −1.19 V vs. Ag/AgCl.

*EIS* was used to assess catalyst activity and distinguish charge‐transfer kinetics from diffusion limitations at the electrode‐electrolyte interface [58, 59, 60, 61]. EIS measurement was performed at −1.19 V vs. Ag/AgCl in 0.5, 1, and 3 m KHCO_3_ electrolytes, saturated with Ar or CO_2_. Figure 5a–d displays Nyquist and Bode magnitude plots, with individual Nyquist plots provided in Figures S18 and S19. The spectra exhibit two semicircles: a small medium‐frequency arc and a dominant low‐frequency arc [62]. The small arcs reflect rapid interfacial processes that do not limit the overall reaction rate, whereas the pronounced low‐frequency arc indicates that processes in this region are related to RDS.

Higher KHCO_3_ concentrations reduce the semicircle radius, particularly under Ar saturation (Figure 5a). At a fixed KHCO_3_ concentration, semicircles are smaller under CO_2_ than under Ar (Figure 5b). The pronounced dependence of the low‐frequency arc on KHCO_3_ concentration and gas environment indicates its association with interfacial charge‐transfer kinetics involving CO_2_ or CO_2_‐derived intermediates. Bode magnitude plots (Figure 5c,d) show the impedance modulus (|Z|) rising from near zero at high frequencies to a maximum at low frequencies, supporting the conclusion that RDS dominates in the low‐frequency region. Under Ar saturation, |Z| at low frequency decreases from 103 to 29 Ω as the bicarbonate concentration increases from 0.5 to 3 m. Meanwhile, the CO_2_‐saturated 1 m KHCO_3_ exhibits a lower |Z| (18 Ω) than Ar (44 Ω) purging condition, reflecting enhanced charge transfer in the presence of CO_2_. Although Nyquist plots exhibit two semicircles under measured conditions, the Bode phase plot (Figure S20) shows a single pronounced peak, likely due to overlapping time constants or a minor contribution from medium‐frequency processes [63]. Potential‐dependent EIS in 1 m KHCO_3_ under Ar saturation (OCP, −1.19 to −1.31 V vs. Ag/AgCl) reveals that the low‐frequency semicircle and |Z| decrease with increasing overpotential (Figure S21), consistent with an accelerated charge transfer RDS. The slow charge‐transfer process observed at low frequencies can be rationalized by coupling to CO_2_ generation via the bicarbonate equilibrium and the adsorption step at Ni single‐atom sites, thereby extending the characteristic time constant [62, 64, 65].

EIS data were fitted using the equivalent circuit model in Figure 5f (parameters in Table S6), employing two parallel R─C elements corresponding to the intermediate‐ and low‐frequency semicircles [66, 67]. The low‐frequency resistance component (*R*_3_), associated with charge transfer and adsorption processes influenced by bicarbonate equilibria, decreases with increasing bicarbonate concentration under both Ar and CO_2_ saturation (Figure 5e). However, *R*_3_ is consistently lower under a CO_2_ atmosphere, highlighting the critical role of CO_2_ in facilitating charge transfer. In 0.5 m KHCO_3_, the difference of *R_3_* (∆*R_3_* = 80 Ω) between CO_2_ and Ar atmosphere is pronounced but becomes smallest at 3 m (16 Ω), indicating sufficient CO_2_ liberation at higher concentrations of KHCO_3_. These combined observations demonstrate that the RDS involving charge transfer is closely linked to CO_2_ availability and chemical equilibria, rather than being a pure interfacial electron‐transfer process.

In summary, the Tafel slopes reveal kinetic limitations arising from electron transfer and buffer capacity. EIS results indicate that the interfacial charge‐transfer resistance is modulated by CO_2_ availability and the applied potential during bicarbonate electroreduction on Ni SAC. The findings support a PE‐ET mechanism (Figure 5g), with electron transfer to adsorbed CO_2_ intermediates as the RDS, governed by dissolved CO_2_ concentration from the bicarbonate equilibrium. The potential‐dependent change in charge transfer resistance confirms the electrochemical nature of the RDS. These insights highlight the importance of optimizing electrolyte composition and operating conditions to improve the efficiency of bicarbonate electrolysis.

## 3 Conclusion

This study elucidates the mechanistic insights and kinetics of bicarbonate electroreduction on a Ni SAC, highlighting the pivotal roles of electrolyte composition and CO_2_(aq) availability. Comparative studies in closed, open, and Ar‐purged cells demonstrate that the depletion of reactive carbon species is the primary cause of selectivity loss in H‐cell configurations. Purging with Ar accelerates the loss of dissolved inorganic carbon [ending up as CO_2_(g)], with CO_2_(aq) decreasing from 0.011 to 0.004 m, raising pH, and lowering CO selectivity. In 3 m KHCO_3_, the Ni SAC achieves a FE_CO_ > 60 %, with FE_CO_ decreasing over time. Infrared spectroscopy reveals this decline in efficiency during prolonged electrolysis due to depletion of CO_2_(aq) and shifts in the bicarbonate‐CO_2_ equilibrium. These effects are mitigated by electrolyte replenishment, electrolyte pH restoration, or closed‐cell operation, which retain CO_2_ and enhance bicarbonate electrolyzer stability. Kinetic analyses, including Tafel slopes (∼118 mV dec^−1^) and reactant‐dependent rates, support a pre‐equilibrium‐coupled electron transfer mechanism. Electrochemical impedance spectroscopy confirms that charge‐transfer resistance is modulated by CO_2_ availability and the electrode potential, with local CO_2_ generation and electron transfer limiting the bicarbonate conversion. Future catalyst design should aim to enhance charge‐transfer kinetics by tuning the Ni‐site electronic structure or coordination environment to improve CO_2_ adsorption/activation. Furthermore, sustained performance requires stable bicarbonate concentration and pH, which can be achieved through periodic electrolyte replenishment or dynamic pH management.

## Author Contributions

Lin Li conducted all electrochemical experiments and infrared spectroscopy analyses, with assistance from Ting Zhang and the guidance of Steen Uttrup Pedersen. The catalysts were synthesized by Yi‐Jie Kong, and Xin‐Ming Hu conceived and supervised the project. Kim Daasbjerg coordinated and supervised the overall project. All authors contributed to the discussion about experimental results.

## Conflicts of Interest

The authors declare no conflicts of interest.

## Supporting information




**Supporting File**: advs74284‐sup‐0001‐SuppMat.pdf.

## Data Availability

The data supporting the findings of this study are available from the corresponding author upon reasonable request.
