# Supplementary material for: Insights Into CO2 Loss, pH Effects, and Tafel Kinetics in Ni Single Atom‐Driven Bicarbonate Electroreduction
Source: Adv Sci (Weinh). 2026 Feb 8:e24353. Online ahead of print. doi: 10.1002/advs.202524353 (PMC13325658; doi:10.1002/advs.202524353)
Supplement: Supplementary file 1 — Supporting File: advs74284‐sup‐0001‐SuppMat.pdf. [file ADVS-9999-e24353-s001.pdf]

Supporting Information

Insights into CO_2 Loss, pH Effects, and Tafel Kinetics in Ni Single Atom-Driven Bicarbonate Electroreduction

Lin Li, Yi-Jie Kong, Ting Zhang, Xu Han, Kristine Aalestrup, Steen Uttrup Pedersen, Xin-Ming Hu, Kim Daasbjerg**

Experimental Section

Chemicals

Potassium bicarbonate ($KHCO_3$), potassium carbonate (K_2CO_3), and potassium chloride (KCl) were supplied by commercial sources (Merck). Nafion^{TM} polymer 5wt% solution was obtained from Ion Power. Isopropanol and hydrochloric acid (HCl) were purchased from Sigma-Aldrich. Dimethyl sulfoxide (99.8%), and deuterium oxide (D 99.9%) were purchased from Cambridge Isotope Laboratories. All reagents were used as received, unless stated otherwise.

Synthesis of Ni SAC

The Ni SAC was synthesized according to the reported procedure.[S1] Firstly, NiO nanoparticles were dispersed in 200 mL of 10 mM tris buffer solution (pH adjusted to 8.5 with 1 M HCl) by ultrasonication. To this suspension, 20 mL of dopamine hydrochloride solution (0.13 M) was added dropwise. The mixture was stirred in air at room temperature for 24 h, then centrifuged. The precipitate was washed three times each with water and ethanol and dried under vacuum at 60 °C, yielding polydopamine-coated NiO nanoparticles (NiO@PDA). The dried material was ground with 0.2 g $NiCl_2 \cdot 6H_2O$ and pyrolyzed at 800 °C in a tube furnace for 2 h under Ar. The resulting solid was washed with 0.5 M H_2SO_4 and dried under vacuum at 60 °C to afford the final Ni SAC. Characterization by AC-HAADF-STEM and XPS confirmed a Ni loading of 2.14 wt% with isolated Ni atoms dispersed on hollow carbon spheres.[S1]

Electrode preparation

Carbon paper (Toray TGP-H-60) was cut into 1 cm × 2 cm pieces and sonicated in acetone and ultrapure water (15 min each). Catalyst ink was prepared by dispersing 6 mg of Ni SAC in 450 μL of ultrapure water and 90 μL of isopropanol, followed by 30 min sonication. Then, 60 μL of a 5 wt% Nafion solution was added, and sonication continued for an additional 30 min. Then, 50 μL of ink was drop-cast onto each side of the carbon paper within a 1 cm^2 active area, yielding a catalyst loading of 0.5 mg cm^{-2}. The electrode was dried at room temperature.

Electrolyzer Configuration and Electrochemical Measurements.

All electrochemical experiments were performed in a custom H-type glass cell separated by a Nafion-117 proton-exchange membrane. The cathode compartment contained the Ni SAC working electrode and an Ag/AgCl (3 M KCl) reference electrode; the anode compartment housed a Pt wire counter electrode. Aqueous electrolyte ($KHCO_3$ or K_2CO_3) with a volume of 33 mL was added to both

chambers before electrolysis. Before each measurement, the cathode electrolyte was purged for 30 min with Ar or CO_2 (flow rate = 30 mL min^{-1}) until saturation. A Bio-Logic electrochemical workstation (SP200) was used for all electrochemical measurements. Potentials were measured against the Ag/AgCl reference electrode (without iR correction). The pH values of the electrolytes under different atmospheres were measured by a pH meter (MeterLab PHM220). The potential versus the reversible hydrogen electrode (RHE) was converted using the following equation:

$$E_{\mathrm{RHE}} = E_{\mathrm{Ag/AgCl}} + 0.197 + 0.059 \times \mathrm{pH}$$

Product Analysis

Gases produced during electrolysis were introduced into the headspace at a flow rate of 30 mL min^{-1}, unless otherwise stated. Gas products (H_2 and CO) were detected and quantified using an online gas chromatograph (SRI Instruments Multiple Gas Analyzer #5) equipped with a thermal conductivity detector (TCD) and flame ionization detector (FID). The liquid product was analyzed by ^{1}H nuclear magnetic resonance (^{1}H NMR, Bruker Ascend Evo). Faradaic efficiency of products (FE_{CO} and FE_{H2}) was calculated as shown below:

$$\mathrm{FE_{product}} = \frac{n \times v \times c \times F \times P}{i \times R \times T}$$

where *n* is the number of electrons required for the given product during the reduction process (*n* = 2), *c* is the volume concentration of the given product in the gas flow, *v* is the gas (Ar or CO_2) flow rate, *i* is the average current at the sampling time, *F* is Faraday's constant (96485 C mol^{-1}), R is the gas constant (8.314 J mol^{-1} K^{-1}), *P* is the atmospheric pressure (101325 Pa), and *T* is the temperature (298.15 K).

The partial current density (j_{CO}) of CO product normalized using the geometric area (A = 2 cm^2):

$$j_{\mathrm{CO}} = \frac{i \times \mathrm{FE_{co}}(\%)}{A}$$

Probing Dissolved CO_2 in Aqueous Electrolyte by Infrared Spectroscopy

Transmission FTIR spectra were recorded on a Bruker INVENIO R spectrometer equipped with a liquid-nitrogen-cooled MCT detector. A demountable, thin-layer, closed-cell liquid comprising a stainless-steel frame, PTFE spacers, and CaF_2 windows was used. The liquid thickness was ~0.05 mm to minimize the effect of water. The cell was placed in the sample compartment throughout the measurements. Samples were injected into the cell via syringe, and the first spectrum at 0 min was

acquired immediately. Time-resolved spectra were collected at defined intervals to monitor signal evolution. Ar-saturated ultrapure water was used as the background, and CO_2-saturated ultrapure water (prepared by bubbling CO_2 for 30 min) was used as the reference for quantification. The $KHCO_3$ aqueous solutions with different concentrations (0.5, 1, and 3 M) were purged with Ar for 30 min (flow rate = 30 mL min^{-1}) before the IR measurements. Based on the consideration of the equilibrium between HCO_3^- and CO_2^{3-} species,[S2] a 3 M K_2CO_3 solution was also analyzed by infrared spectroscopy under the same conditions. Spectra were processed in OPUS software. Baseline correction was performed using polynomial fitting followed by rubberband correction. The same correction parameters were used for all spectra to ensure comparability of relative band intensities.

pH Measurements

To investigate the effect of gas–liquid exchange on carbonate speciation and pH evolution, the time-dependent pH of 3 M $KHCO_3$ was monitored over 4 h (30 min intervals) in two systems with no electrolysis applied, i.e., i) Open cell, where the initial pH was recorded immediately after totally dissolving $KHCO_3$ in deionized water. ii) Ar-purged open cell, where Ar was first bubbled through the solution for 30 min and then redirected to the headspace for 4 h to maintain an Ar atmosphere. Note that the Ar-purged open system simulates the electrolysis experiment, but without applying a potential.

Electrochemical Impedance Spectroscopy Measurements and Fitting Details.

EIS measurements were performed at a fixed potential of −1.19 V vs Ag/AgCl, corresponding to the Tafel analysis region. The measurements were carried out in $KHCO_3$ solutions of varying concentrations (0.5−3.0 M) under both CO_2- and Ar-saturated atmospheres (30 min of purging). The frequency range was swept from 3 kHz to 0.01 Hz with an amplitude of 5 mV. Additionally, potential-dependent impedance was recorded in Ar-saturated 1 M $KHCO_3$. All measurements were performed without iR compensation unless otherwise specified.

Impedance data were fitted using the Z-fit module in EC-lab, simultaneously fitting the real and imaginary components to extract circuit parameters. The fit quality was evaluated by comparing the experimental and simulated Nyquist plots**.** The equivalent circuit was selected based on the semicircular shape of Nyquist plots observed under the tested conditions.

Supplementary Figures and Tables

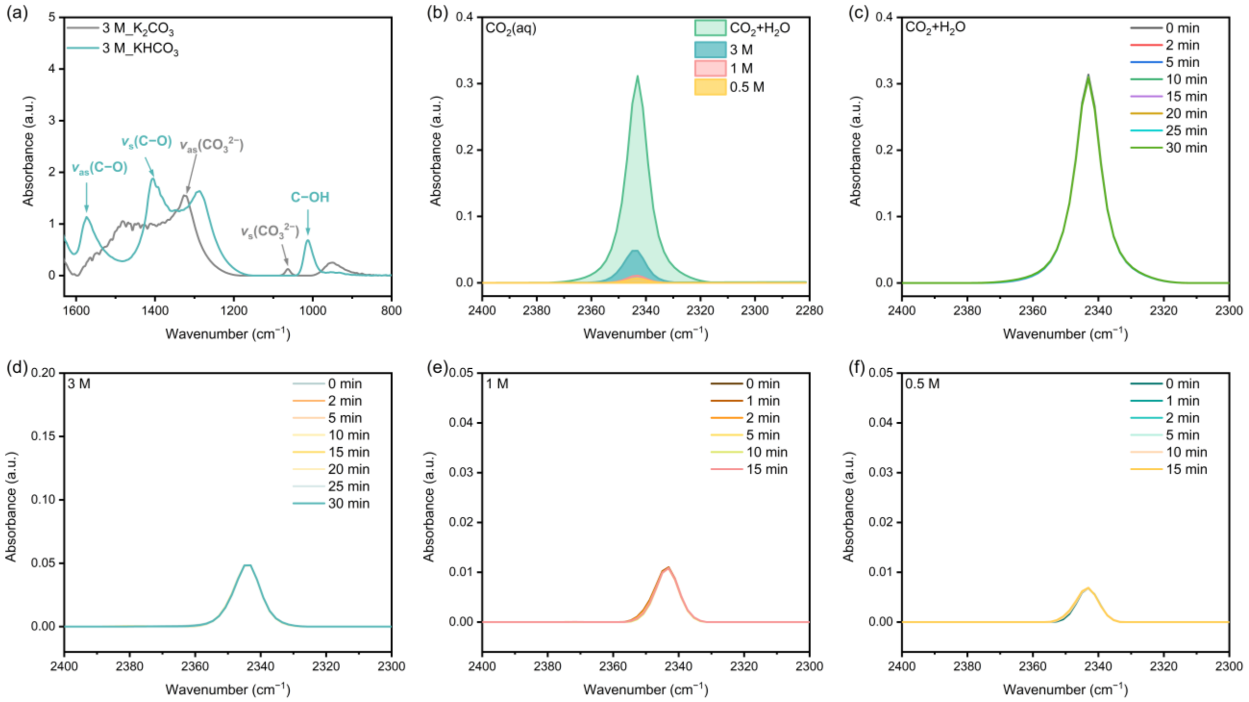

Figure S1. (a) IR peaks attributed to bicarbonate and carbonate species. (b) Integrated peak areas of the CO_2(aq) spectra. Time-dependent infrared responses of dissolved CO_2 in (c) CO_2-saturated solution and in Ar-saturated $KHCO_3$ solutions with varying concentrations of (d) 3 M, (e) 1 M, and (f) 0.5 M.

No IR signal of CO_2(aq) is detected in 3 M K_2CO_3 solution, while a prominent absorption band at 1327 cm^{-1} and a peak at 1064 cm^{-1} (**Figure S1a**) corresponding to characteristic vibrations of carbonate species.[S3] Using the known CO_2 concentration in CO_2-saturated water (33 mM) as a reference,[S4] the concentration of dissolved CO_2 is determined from the peak area (**Figure S1b**).

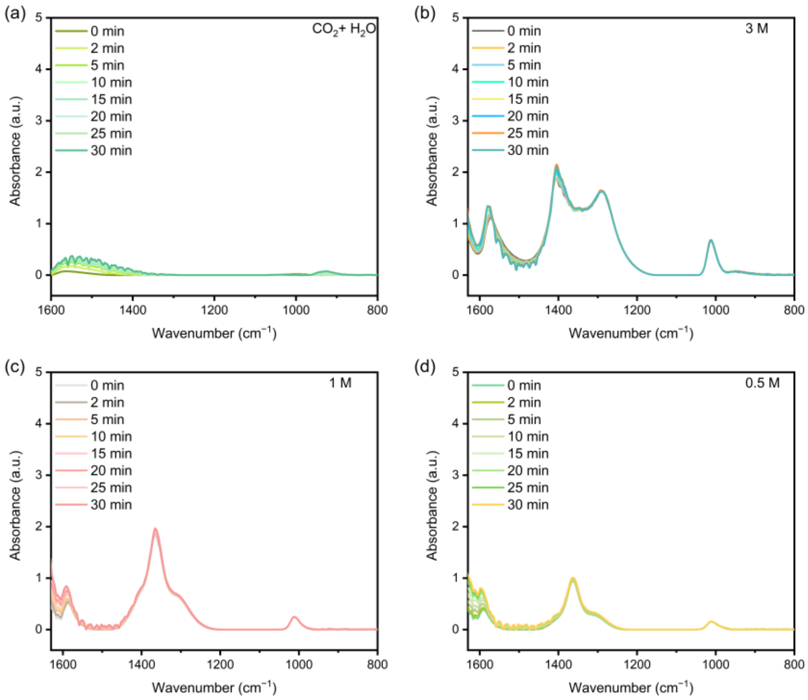

Figure S2. IR peaks attributed to bicarbonate and carbonate species in different solutions: (a) CO_2-saturated H_2O and Ar-saturated (b) 3 M, (c) 1 M, and (d) 0.5 M $KHCO_3$.

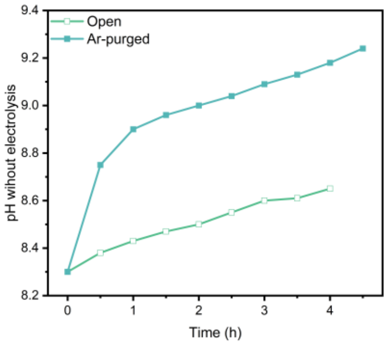

Figure S3. Increase in pH of 3 M $KHCO_3$ solution over time for the open-cell and Ar-purged open-cell configurations in the absence of electrolysis.

Figure S3 shows that the pH increases in both cases, with the increase most pronounced under Ar purging (8.33 → 9.24 over 4.5 h). Ar purging removes dissolved CO_2, shifting equilibria toward proton consumption and alkalisation, especially the first 1 h. In an open cell, the pH of the $KHCO_3$ solution also increases, albeit more slowly, due to a slower equilibration with the low atmospheric CO_2 partial pressure. These results confirm that the spontaneous pH rise, driven primarily by CO_2 removal, is the dominant factor reducing the availability of CO_2(aq).

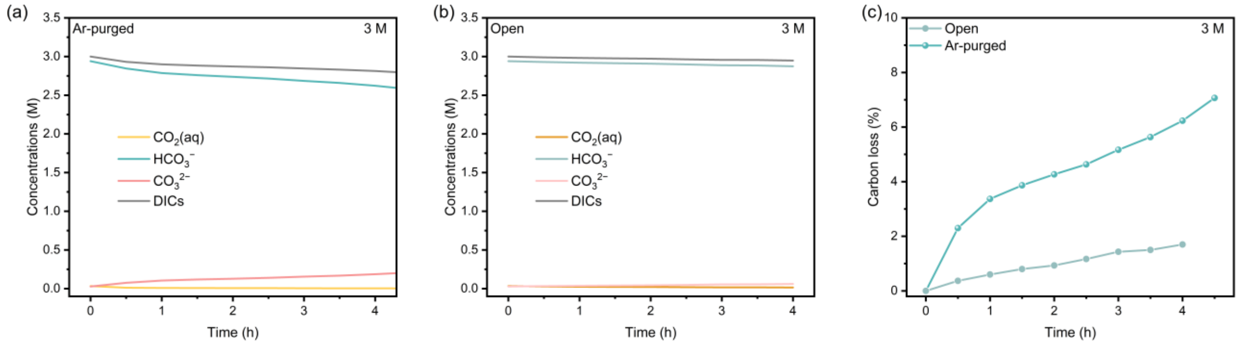

Figure S4. Concentrations of DIC species over time under (a) Ar-purged open cell and (b) open cell in 3 M $KHCO_3$. (c) Percentage of carbon loss over time for both cell configurations in 3 M $KHCO_3$. Data in this figure were computed according to the theoretical model described below.

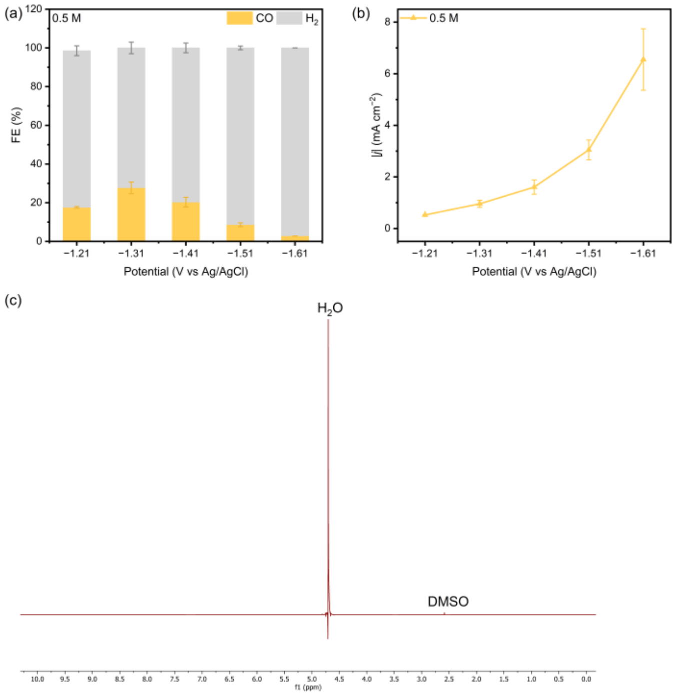

Figure S5. (a,b) Electrochemical performance for bicarbonate reduction using a Ni-SAC in Ar-purged 0.5 M $KHCO_3$ electrolyte. (c) ^{1}H NMR results of the electrolyte after electrolysis.

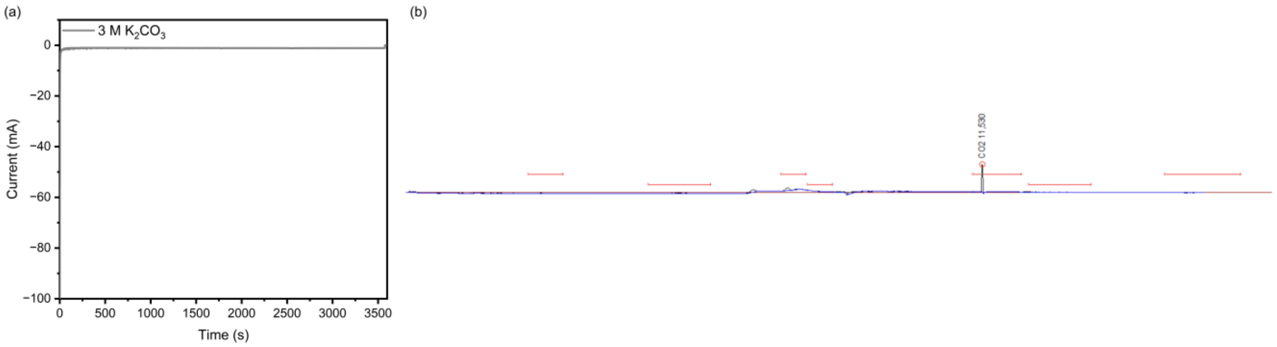

Figure S6. (a) Plot of *i*-t curve and corresponding (b) GC-FID chromatograms obtained during electrolysis using a Ni-SAC at –1.31 V vs Ag/AgCl in Ar-saturated 3 M K_2CO_3.

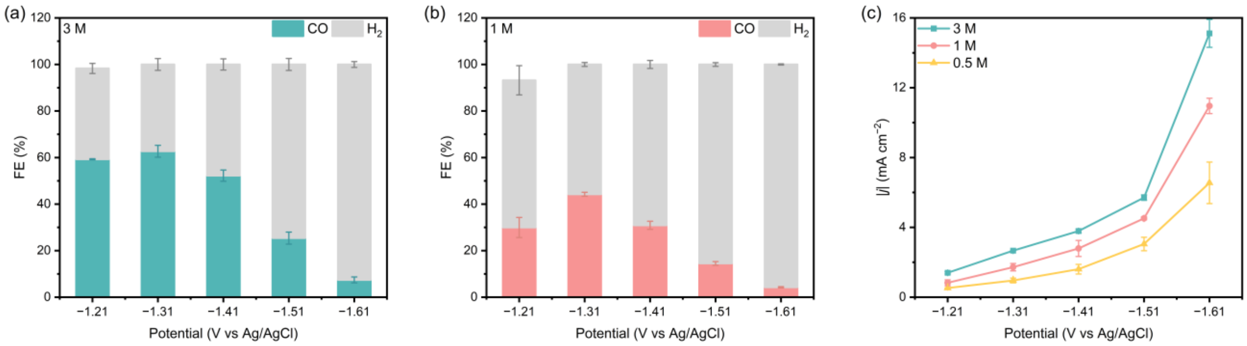

Figure S7. FE of products using a Ni SAC catalyst at progressively more negative potentials in Ar-saturated (a) 3 M and (b) 1 M $KHCO_3$. (c) |*j*| at progressively more negative potentials in $KHCO_3$ electrolyte ot varying concentrations (0.5, 1, and 3 M).

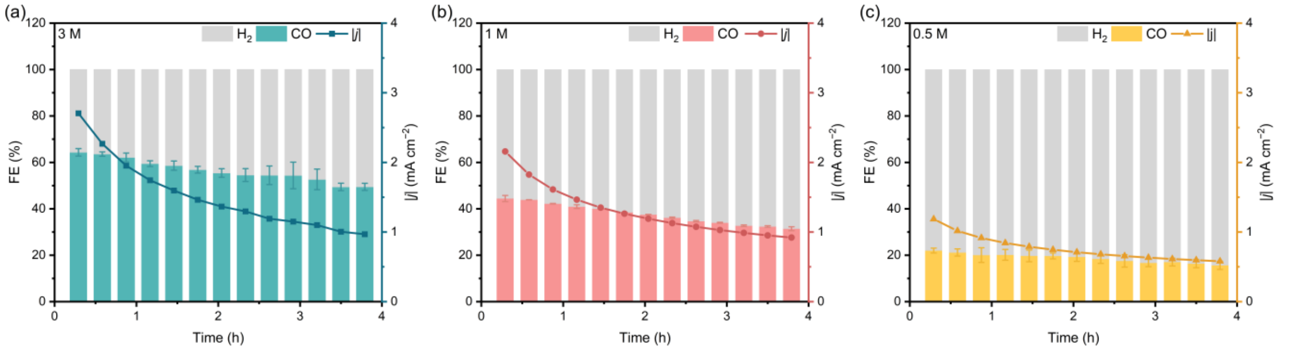

Figure S8. FE and |*j*| from 4 h electrolysis using a Ni SAC at −1.31 V vs Ag/AgCl in Ar-saturated (a) 3 M, (b) 1 M, and (c) 0.5 M $KHCO_3$.

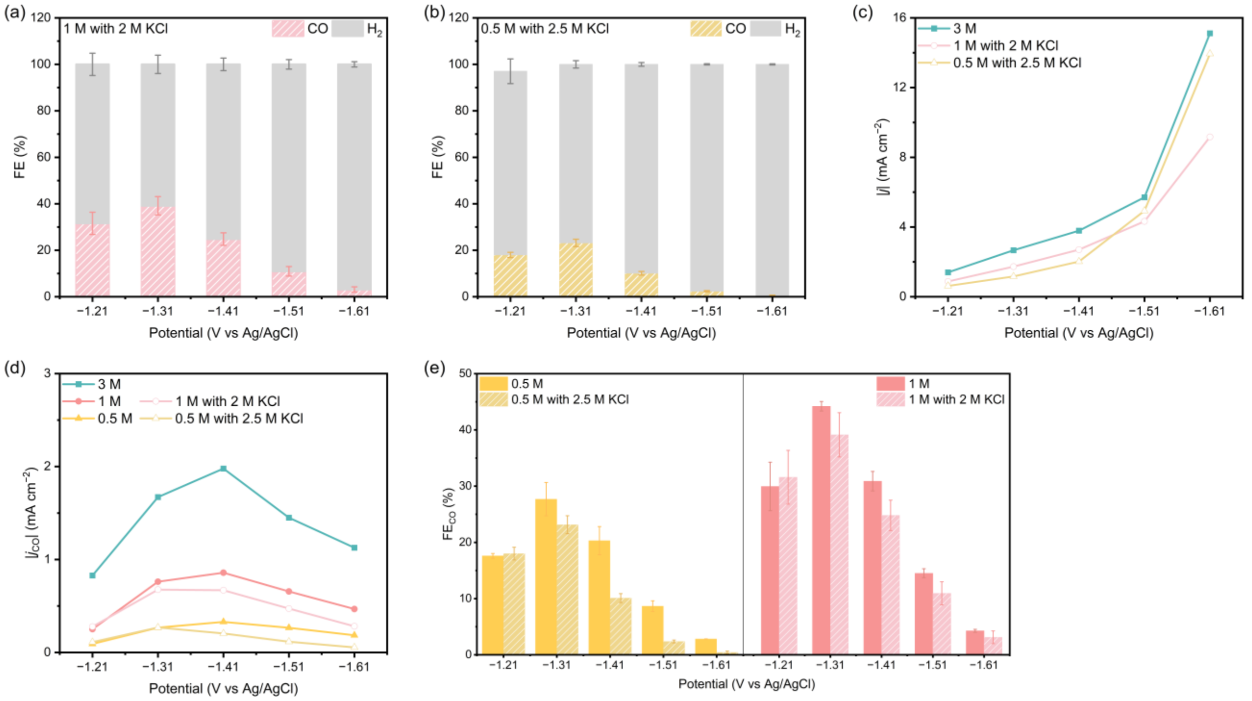

Figure S9. Electrochemical performance using a Ni SAC in Ar-saturated $KHCO_3$ electrolyte with KCl added: (a) 1 M $KHCO_3$ + 2 M KCl and (b) 0.5 M $KHCO_3$ + 2.5 M KCl at progressively more negative potentials, comparison of (c) |*j*| and (d) |j_{CO}|, and (e) FE_{CO} of electrolyte systems with and without KCl added.

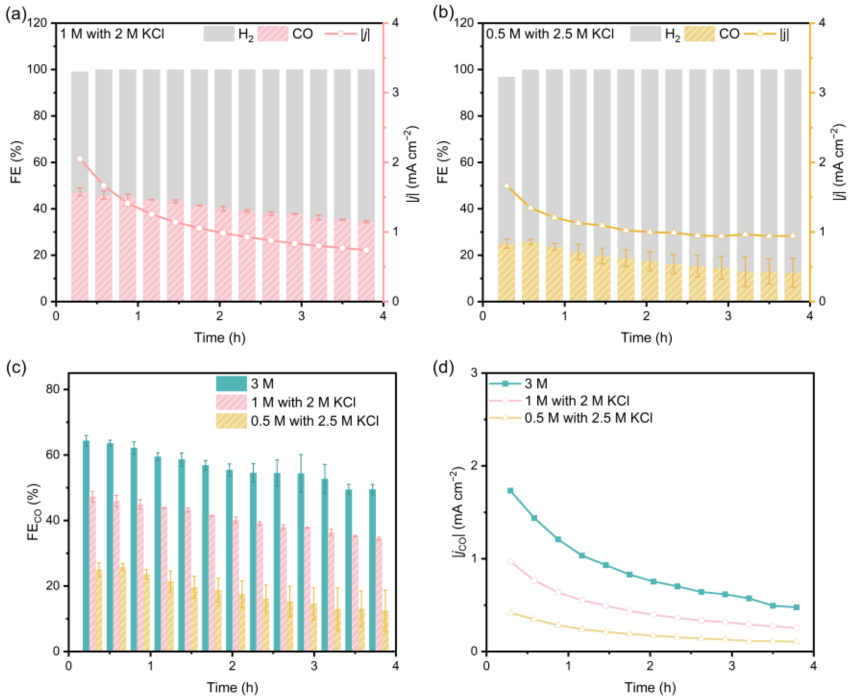

Figure S10. FE_{CO} and $|j|$ from 4 h electrolysis using a Ni SAC at −1.31 V vs Ag/AgCl in Ar-saturated $KHCO_3$ with KCl added: (a) 1 M $KHCO_3$ + 2 M KCl and (b) 0.5 M $KHCO_3$ + 2.5 M KCl. (c,d) Comparison with results from 3 M $KHCO_3$.

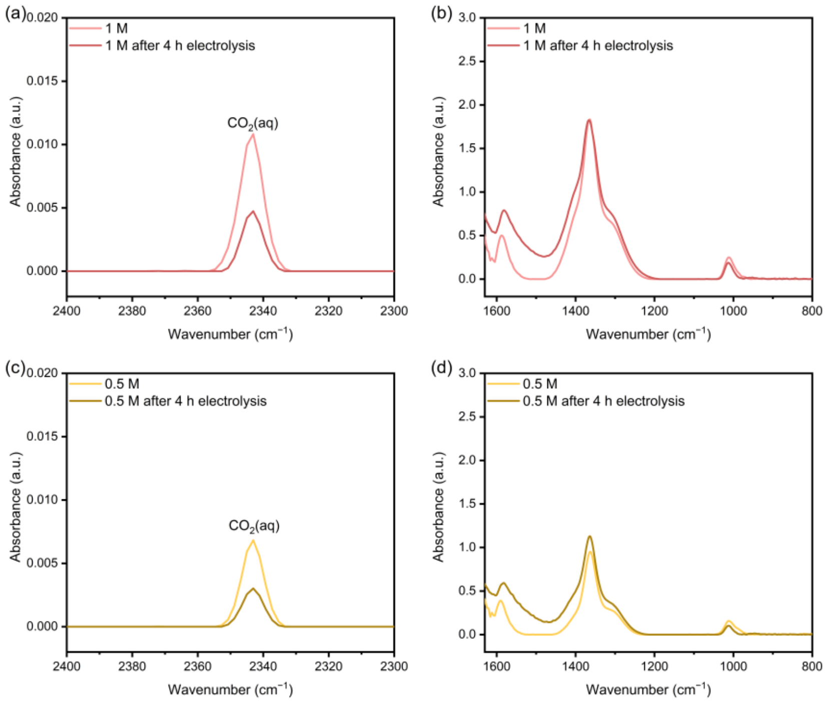

Figure S11. IR signals corresponding to dissolved CO_2, bicarbonate, and carbonate species in Ar-saturated electrolyte before and after 4 h electrolysis using a Ni SAC: (a,b) 1 M and (c,d) 0.5 M $KHCO_3$.

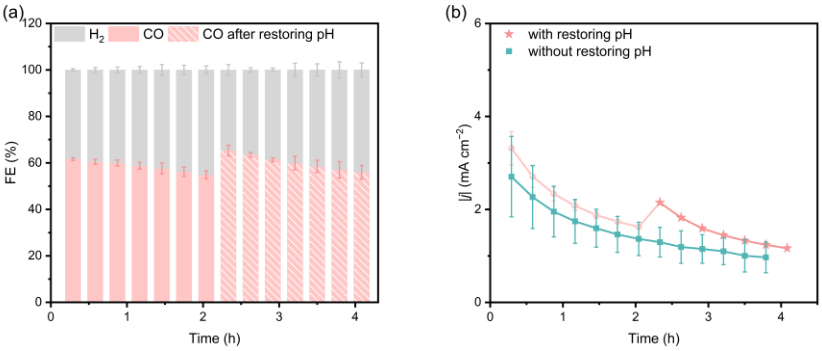

Figure S12. (a) FE and (d) |*j*| before and after pH adjustment during 4 h electrolysis at −1.31 V vs Ag/AgCl.

Hydrochloric acid (HCl, 1 M) served as the external proton source. The initial pH of the 3 M $KHCO_3$ electrolyte was 8.36. After 2 h of electrolysis at −1.31 V vs Ag/AgCl, the pH rose to 9.19. The addition of 9 mL of 1 M HCl (9×10^{-3} mol H^+) restored pH to 8.33. The cell was purged with Ar for 15 min, and electrolysis was resumed for an additional 2 h under identical conditions, yielding a final pH = 9.13.

After pH restoration, FE_{CO} declined over time, mirroring the trend observed in the initial 2 h (**Figure S12**). This behaviour arises from a gradual increase in pH during continued electrolysis, which reduces the availability of CO_2(aq) in the 3 M $KHCO_3$ electrolyte. These results demonstrate that rising pH depletes the reactive CO_2(aq) in the electrolyte, thereby constituting the primary cause of the loss of CO production. Although irreversible catalyst deactivation cannot be entirely excluded, the dominant factor limiting performance over time remains the diminished availability of CO_2(aq).

Additionally, the amount of HCl required for pH restoration exceeded the quantity expected from simple neutralization owing to the buffering capacity of the carbonate/bicarbonate system:

$$\mathrm{pH} = \mathrm{p}K_2 + \log\frac{[\mathrm{CO_3^{2-}}]}{[\mathrm{HCO_3^-}]}$$

The Henderson-Hasselbalch relationship shows that pH is governed by the ratio of carbonate and bicarbonate concentrations, rather than the local acid or base added. Added H^+ first converts CO_3^{2-} to HCO_3^- and subsequently protonates HCO_3^- to form H_2CO_3, which decomposes to CO_2(aq) + H_2O. In an open system, partial CO_2 degassing shifts the equilibrium, necessitating the addition of excess acid to reestablish the target $[CO_3^{2-}]/[HCO_3^-]$ ratio corresponding to pH ≈ 8.3.

During the first 2 h of electrolysis, a total of 32.514 Coulombs was passed, driving CO and H_2 production via the reactions:

$$CO_2 + 2H^+ + 2e^- \rightarrow CO + 2H_2O$$

$$2H^+ + 2e^- \rightarrow H_2$$

The amount of produced CO can be calculated:

$$n_{CO} = \frac{FE_{CO} \times Q_{total}}{n \times F} = \frac{57.5\% \times 32.514\ C}{2 \times 96485\ C\ mol^{-1}} = 9.69 \times 10^{-5}\ mol$$

where n is the molar amount of the corresponding product, z is the number of electrons transferred per molecule of product (2 for CO), and Q_{total} is the total charge passed during electrolysis. FE_{CO} = 57.5% is the average during the first 2 h of electrolysis. Based on the above, the [DIC] consumed by electrolysis was ~0.003 M (electrolyte volume = 33 mL).

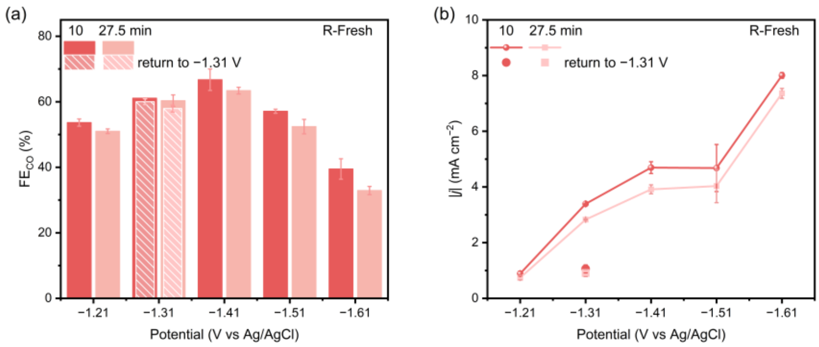

Figure S13. FE_{CO} and $|j|$ measured at 10 and 27.5 min of electrolysis using a Ni SAC at progressively more negative potentials in an Ar-purged H-cell under R-Fresh condition; slashed bars and discrete points represent the results measured upon returning to −1.31 V vs Ag/AgCl.

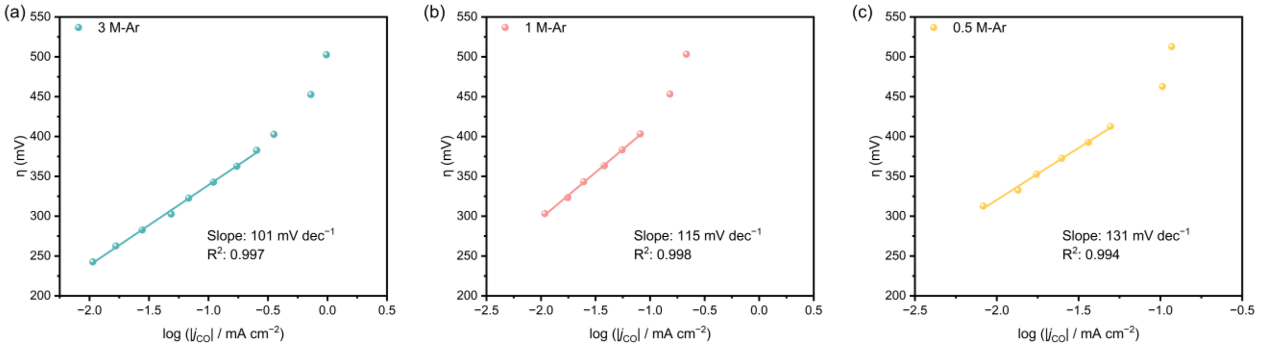

Figure S14. Tafel plots of bicarbonate conversion using a Ni SAC in Ar-saturated $KHCO_3$ at varying concentrations: (a) 3, (b) 1, and (c) 0.5 M. The coefficient of determination (R^2) for the linear fits is included in each plot.

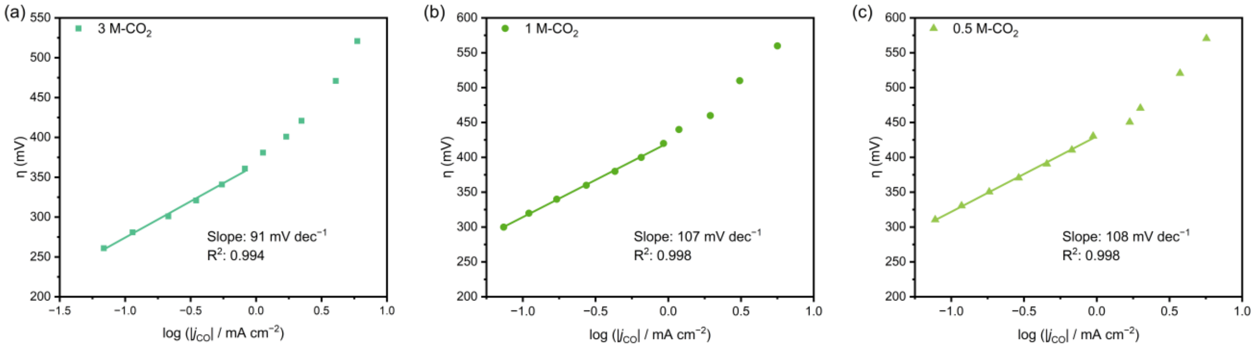

Figure S15. Tafel plots of eCO_2RR using a Ni SAC in CO_2-saturated $KHCO_3$ electrolyte of varying concentrations: (a) 3, (b) 1, and (c) 0.5 M. The coefficient of determination (R^2) for the linear fits is included in each plot.

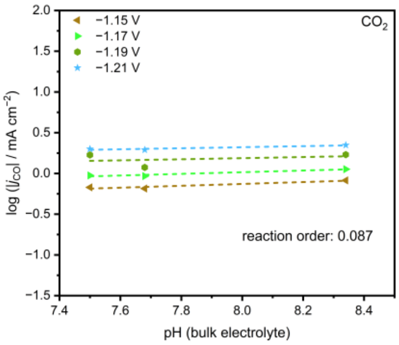

Figure S16. Plot of log $|j_{CO}|$ vs bulk pH at applied potentials of −1.15, −1.17, −1.19, and −1.21 V vs Ag/AgCl under CO_2-saturated conditions.

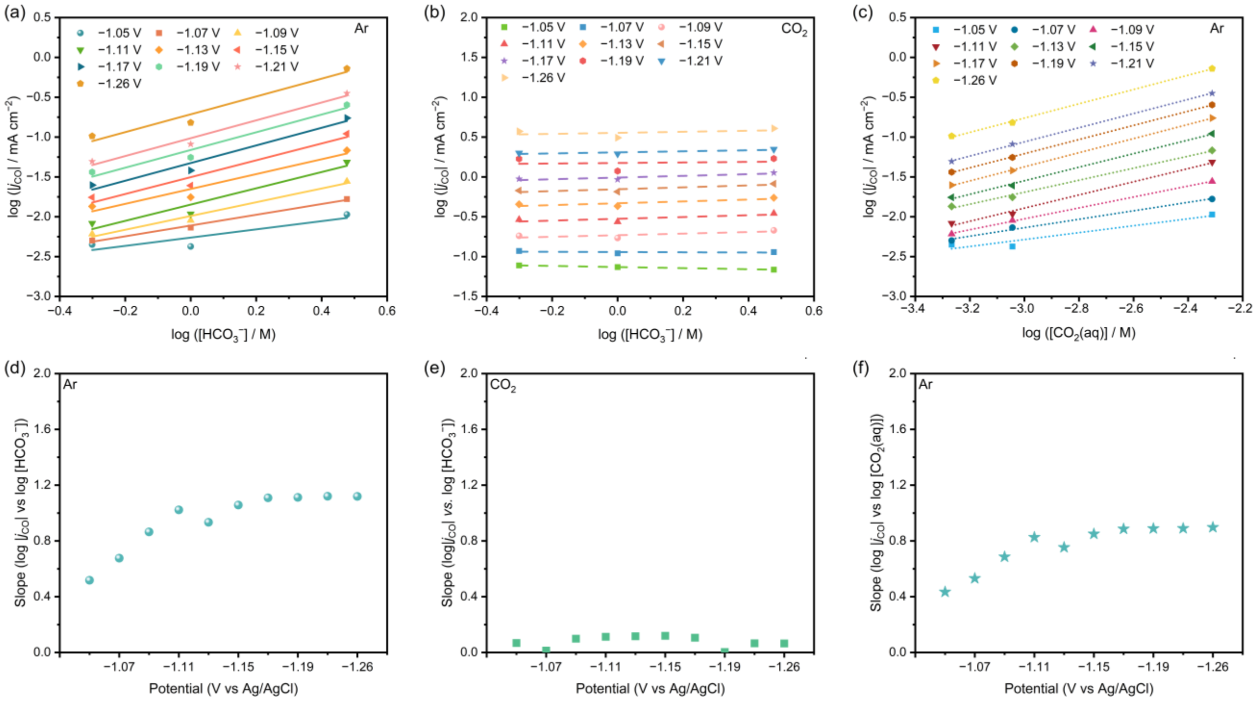

Figure S17. Dependence of |j_{CO}| on the concentration of bicarbonate under (a) Ar (bicarbonate conversion) and (b) CO_2 (eCO_2RR). (c) Dissolved CO_2 dependence for bicarbonate conversion. (d-f) Slopes extracted from the log-log plots in (a-c) at varying potentials.

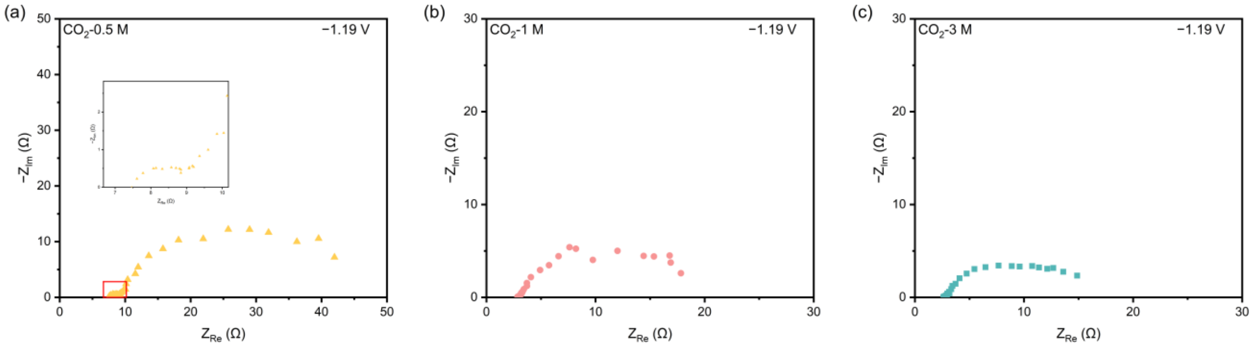

Figure S18. Nyquist plots obtained using a Ni-SAC in CO_2-saturated $KHCO_3$ electrolyte at varying concentrations: (a) 0.5 (inset is zoomed-in area of plot), (b) 1, and (c) 3 M.

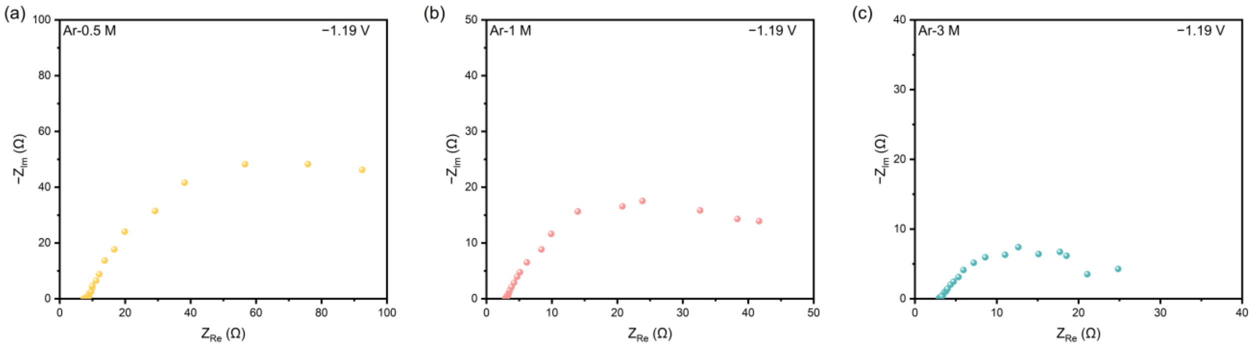

Figure S19. Nyquist plots obtained using a Ni-SAC in Ar-saturated $KHCO_3$ electrolyte of varying concentrations: (a) 0.5 M, (b) 1 M and (c) 3 M.

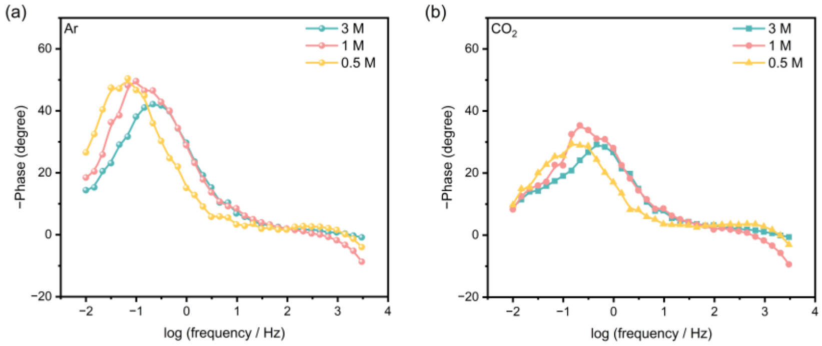

Figure S20. Bode phase plots of Ni-SAC in (a) Ar- and (b) CO_2-saturated $KHCO_3$ at −1.19 V vs Ag/AgCl, showing phase angle vs logarithmic frequency measured with an AC amplitude of 5 mV and frequency of 3 kHz–0.01 Hz.

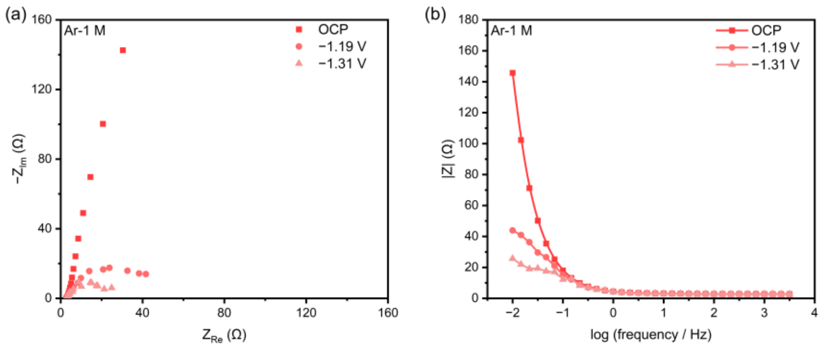

Figure S21. (a) Nyquist plots and (b) the corresponding Bode magnitude plots obtained using a Ni-SAC in Ar-saturated 1 M $KHCO_3$ at –1.19 and –1.31 V vs Ag/AgCl.

Table S1. Quantification of Aqueous CO_2(aq) in Various Solutions (Closed Cell) after 30 min Ar Purging.

Solution	pH	Area (a.u.)	[CO_2(aq)] (mM)
$CO_2 + H_2O$	-	3.632	33.000
0.5 M $KHCO_3$	8.48	0.056	0.513
1.0 M $KHCO_3$	8.64	0.098	0.893
3.0 M $KHCO_3$	8.75	0.537	4.880

Table S2. pH Changes of 3 M $KHCO_3$ in Open Cell without Electrolysis and Calculated Concentrations of Carbon Species.[a]

Time (min)	pH	[CO_2(aq)] (M)	[HCO_3^-] (M)	[CO_3^{2-}] (M)	[DIC] (M)	Carbon Loss (M)
Initial[b]	8.3	0.033	2.940	0.027	3.000	0.000
30	8.38	0.027	2.929	0.033	2.989	0.011
60	8.43	0.024	2.921	0.037	2.982	0.018
90	8.47	0.022	2.914	0.040	2.976	0.024
120	8.5	0.021	2.908	0.043	2.972	0.028
150	8.55	0.018	2.898	0.048	2.965	0.035
180	8.6	0.016	2.887	0.054	2.957	0.043
210	8.61	0.016	2.885	0.055	2.955	0.045
240	8.65	0.014	2.874	0.060	2.949	0.051

[a] Concentrations of carbon species were computed according to the theoretical framework described herein. [b] Measurement starts after ensuring complete dissolution of $KHCO_3$.

Table S3. pH Changes of 3 M $KHCO_3$ in Ar-purged Open Cell without Electrolysis and Calculated Concentrations of Carbon Species.[a]

Time (min.)	pH	$[CO_2(aq)]$ (M)	$[HCO_3^-]$ (M)	$[CO_3^{2-}]$ (M)	[DIC] (M)	Carbon Loss (M)
Initial[b]	8.3	0.033	2.940	0.027	3.000	0.000
30	8.75	0.011	2.845	0.075	2.931	0.069
60	8.9	0.008	2.787	0.104	2.899	0.101
90	8.96	0.007	2.759	0.118	2.884	0.116
120	9.00	0.006	2.738	0.128	2.872	0.128
150	9.04	0.006	2.716	0.139	2.861	0.139
180	9.09	0.005	2.685	0.155	2.845	0.155
210	9.13	0.004	2.659	0.168	2.831	0.169
240	9.18	0.004	2.623	0.186	2.813	0.187
270	9.24	0.003	2.576	0.209	2.788	0.212

[a] Concentrations of carbon species were computed according to the theoretical framework described herein. [b] Measurement starts after ensuring complete dissolution of $KHCO_3$.

Table S4. Possible eCO_2RR Mechanisms with Corresponding Tafel Slopes and Rate Expressions.[S5-6] [a]

	RDS of proposed mechanism	Tafel Slope mV decade^{-1}	Rate expression
A1	$\theta_{\mathrm{M}} + \mathrm{CO_2} + \mathrm{e^-} \rightleftharpoons \theta_{\mathrm{MCO_2^-}}$	118	$j_{\mathrm{CO}} = FAk^0_{\mathrm{A1}}\theta_{\mathrm{M}}[\mathrm{CO_2}]\exp\left(-\frac{\beta F\eta}{RT}\right)$
A2	$\theta_{\mathrm{MCO_2^-}} + \mathrm{H^+} \rightleftharpoons \theta_{\mathrm{MCOOH}}$	59	$j_{\mathrm{CO}} = FAk^0_{\mathrm{A2}}[\mathrm{CO_2}][\mathrm{H^+}]\exp\left(-\frac{F\eta}{RT}\right)$
A3	$\theta_{\mathrm{MCOOH}} + \mathrm{H^+} + \mathrm{e^-} \rightleftharpoons \mathrm{CO_{ad}} + \mathrm{H_2O}$	39	$j_{\mathrm{CO}} = FAk^0_{\mathrm{A3}}K_{A2}[\mathrm{CO_2}][\mathrm{H^+}]^2\exp\left(-\frac{(1+\beta)F\eta}{RT}\right)$
B1	$\theta_{\mathrm{M}} + \mathrm{CO_2} + \mathrm{H^+} + \mathrm{e^-} \rightleftharpoons \theta_{\mathrm{MCOOH}}$	118	$j_{\mathrm{CO}} = FAk^0_{\mathrm{B1}}\theta_{M}[\mathrm{CO_2}][\mathrm{H^+}]\exp\left(-\frac{\beta F\eta}{RT}\right)$
B2	$\theta_{\mathrm{MCOOH}} + \mathrm{H^+} + \mathrm{e^-} \rightleftharpoons \mathrm{CO_{ad}} + \mathrm{H_2O}$	39	$j_{\mathrm{CO}} = FAk^0_{\mathrm{B2}}[\mathrm{CO_2}][\mathrm{H^+}]^2\exp\left(-\frac{(1+\beta)F\eta}{RT}\right)$
C1	$\theta_{\mathrm{M}} + \mathrm{CO_2} + \mathrm{H^+} + \mathrm{e^-} \rightleftharpoons \theta_{\mathrm{MCOOH}}$	118	$j_{\mathrm{CO}} = FAk^0_{\mathrm{C1}}\theta_{M}[\mathrm{CO_2}][\mathrm{H^+}]\exp\left(-\frac{\beta F\eta}{RT}\right)$
C2	$\theta_{\mathrm{MCOOH}} + \mathrm{H^+} \rightleftharpoons \theta_{\mathrm{MCOOH-H}}$	59	$j_{\mathrm{CO}} = FAk^0_{\mathrm{C2}}[\mathrm{CO_2}][\mathrm{H^+}]^2\exp\left(-\frac{F\eta}{RT}\right)$
C3	$\theta_{\mathrm{MCOOH-H}} + \mathrm{e^-} \rightleftharpoons \mathrm{CO_{ad}} + \mathrm{H_2O}$	39	$j_{\mathrm{CO}} = FAk^0_{\mathrm{C3}}K_{\mathrm{C2}}[\mathrm{CO_2}][\mathrm{H^+}]^2\exp\left(-\frac{(1+\beta)F\eta}{RT}\right)$

[a] θ_M denotes an active site on the electrode surface, H^+ represents the proton donor which may be H_2O or HCO_3^-, β is assumed to be 0.5, F is Faraday's constant (96485.34 C mol^{-1}), R is the gas constant (8.314 J mol^{-1} K^{-1}), and T is the temperature (298.15 K).

Table S5. Reaction Order Slopes Obtained from Log-Log Plots (Linear Fits) as Function of Potential.[a]

Potentials (V vs Ag/AgCl)	Slopes		
	Ar: log $\lvert j_{CO}\rvert$ vs log ($[HCO_3^-]$)	Ar: log $\lvert j_{CO}\rvert$ vs log ($[CO_2(aq)]$)	CO_2: log $\lvert j_{CO}\rvert$ vs log ($[HCO_3^-]$)
−1.05	0.518	0.434	0.068
−1.07	0.676	0.531	0.013
−1.09	0.864	0.686	0.099
−1.11	1.023	0.826	0.112
−1.13	0.934	0.753	0.116
−1.15	1.057	0.850	0.119
−1.17	1.109	0.886	0.106
−1.19	1.120	0.889	0.003
−1.21	1.120	0.890	0.066
−1.26	1.119	0.897	0.065

[a] Data show the dependence of CO production on reactant concentration under Ar (bicarbonate conversion) and CO_2 (eCO_2RR).

Table S6. Resistances Obtained from EIS in $KHCO_3$ at Varying Concentrations.

Electrolyte	Ar-saturated			CO_2-saturated		
Resistance (Ohm)	R_1	R_2	R_3	R_1	R_2	R_3
3 M	2.84	0.18	27.35	2.63	0.16	11.71
1 M	2.90	0.34	43.47	2.86	0.13	16.25
0.5 M	7.62	1.01	116.50	7.69	1.28	36.80

Theoretical Framework for Bicarbonate Equilibria

Equilibria in Bicarbonate Aqueous Solution

Following the theoretical framework reported in previous studies,[S7-8] we applied the established principles to investigate the change of the bicarbonate solution during gas purging. In Ar-saturated aqueous $KHCO_3$ solutions, four dissolved inorganic carbon (DIC) species coexist due to pH-dependent equilibria, i.e., CO_2(aq), HCO_3^-, CO_3^{2-}, and H_2CO_3 (eqs (S1−S4), with equilibrium constant at 25 °C).[S9-10]

$$\mathrm{CO_2(aq) + H_2O \rightleftharpoons HCO_3^- + H^+} \qquad (K_1 = 4.47 \times 10^{-7},\ \mathrm{p}K_1 = 6.35) \qquad \text{(S1)}$$

$$\mathrm{HCO_3^- \rightleftharpoons CO_3^{2-} + H^+} \qquad (K_2 = 4.69 \times 10^{-11},\ \mathrm{p}K_1 = 10.33) \qquad \text{(S2)}$$

$$\mathrm{H_2O \rightleftharpoons H^+ + OH^-} \qquad (K_\mathrm{w} = 10^{-14}) \qquad \text{(S3)}$$

$$\mathrm{CO_2(aq) \rightleftharpoons CO_2(g)} \qquad \text{(S4)}$$

The concentration of H_2CO_3 is negligible because it rapidly decomposes to CO_2(aq) in water (~20 s^{-1} at 25 °C).[S11-12] The speciation is governed by bulk pH and total inorganic carbon concentration. In the absence of external CO_2 pressure, the measured [CO_2(aq)] is considerably lower than in CO_2-saturated water (0.033 M at 1 atm, 25 °C)[S4,13] because of the escape of CO_2 to the atmosphere (eq (S4)).

The equilibrium constants K_1, K_2, and K_w are related to concentrations as given in eqs (S5–S7).

$$K_1 = \frac{[\mathrm{HCO_3^-}][\mathrm{H^+}]}{[\mathrm{CO_2(aq)}]} \qquad \text{(S5)}$$

$$K_2 = \frac{[\mathrm{CO_3^{2-}}][\mathrm{H^+}]}{[\mathrm{HCO_3^-}]} \qquad \text{(S6)}$$

$$K_\mathrm{w} = [\mathrm{H^+}][\mathrm{OH^-}] \qquad \text{(S7)}$$

The mass balance of DIC is given by eq (S8).

$$[\mathrm{DIC}] = [\mathrm{CO_2(aq)}] + [\mathrm{HCO_3^-}] + [\mathrm{CO_3^{2-}}] = (\alpha_0 + \alpha_1 + \alpha_2)[\mathrm{DIC}] \qquad \text{(S8)}$$

where α_0, α_1, and α_2 are corresponded to the molar ratio of [CO_2(aq)], [HCO_3^-], and [CO_3^{2-}], respectively.

The alkalinity of the bicarbonate system is defined as in eq (S9).

$$\text{Alkalinity} = [HCO_3^-] + 2[CO_3^{2-}] + [OH^-] - [H^+] = (\alpha_1 + 2\alpha_2)[DIC] + \frac{K_w}{[H^+]} - [H^+] \quad (S9)$$

The concentrations of CO_2(aq), HCO_3^- and CO_3^{2-} can be expressed as functions of [DIC] and [H^+] according to eqs (S10–S12).

$$\alpha_0 = \frac{[CO_2(aq)]}{[DIC]} = 1/\left(1 + \frac{K_1}{[H^+]} + \frac{K_1K_2}{[H^+]^2}\right) \quad (S10)$$

$$\alpha_1 = \frac{[HCO_3^-]}{[DIC]} = 1/\left(1 + \frac{[H^+]}{K_1} + \frac{K_2}{[H^+]}\right) \quad (S11)$$

$$\alpha_2 = \frac{[CO_3^{2-}]}{[DIC]} = 1/\left(1 + \frac{[H^+]}{K_2} + \frac{[H^+]^2}{K_1K_2}\right) \quad (S12)$$

When the system reaches to the equilibria (eqs S5 and (S6)), the mass balance (eq (S8), and the charge balance (eq (S9) constitute six unknown variables ([DIC], [CO_2(aq)], [HCO_3^-], [CO_3^{2-}], [H^+], Alkalinity). Therefore, the concentrations of other carbon species components can be calculated when two variables are known.[[S14]] For instance, in $KHCO_3$ solution without external acid or base, [H^+] (i.e., pH) can be directly measured and the fixed Alkalinity equals [K^+], the theoretical concentrations of dissolved carbon species can be calculated based on eqs (S10)–(S12).

Computational Details

Calculation conditions:

- Alkalinity (A) is assumed to be constant. Only CO_2(aq), HCO_3^-, CO_3^{2-} considered.
- Temperature: 25 °C.
- Equilibrium constants pK_1 = 6.35, pK_2 = 10.33, and pK_W = 14.
- Initial [DIC] = 3 M, initial pH = 8.3.
- Activity coefficients = 1 (ideal solution).
- Local chemical equilibrium reached at each measured pH.

Python Code to calculate the theoretical concentration of DIC species.

```
import math
pKa1 = 6.35
pKa2 = 10.33
Kw = 1e-14
CT0 = 3
```

```
pH0 = 8.3
pH1 =   # the measured pH

def fractions (pH, pKa1, pKa2):
    denom = 1 + 10**(pH - pKa1) + 10**(2*pH - (pKa1+pKa2))
    alpha0 = 1 / denom
    alpha1 = 10**(pH - pKa1) / denom
    alpha2 = 10**(2*pH - (pKa1+pKa2)) / denom
    return alpha0, alpha1, alpha2

def alkalinity(CT, pH, pKa1, pKa2):
    H = 10**(-pH)
    OH = Kw / H
    alpha0, alpha1, alpha2 = fractions(pH, pKa1, pKa2)
    return (alpha1 + 2*alpha2) * CT + (OH - H)

A0 = alkalinity(CT0, pH0, pKa1, pKa2)

alpha0_1, alpha1_1, alpha2_1 = fractions(pH1, pKa1, pKa2)
H1 = 10**(-pH1)
OH1 = Kw / H1
CT1 = (A0 - (OH1 - H1)) / (alpha1_1 + 2*alpha2_1)

deltaC = CT0 - CT1
loss_percent = deltaC / CT0 * 100
CO2 = alpha0_1 * CT1
HCO3 = alpha1_1 * CT1
CO3 = alpha2_1 * CT1

print("initial alkalinity A0 =", A0, "M")
print("DIC CT1 =", CT1, "M")
print("carbon loss ΔC =", deltaC, "M")
print("loss_percent =", loss_percent, "%")
print(f"[CO2(aq)] =", CO2, "M")
print(f"[HCO3⁻] =", HCO3, "M")
print(f"[CO32⁻] =", CO3, "M")
```

Tafel Analysis

The linear relationship between overpotential (η) and the current density (j) is described by the Tafel equation (eq (S13)).[S15]

$$\eta = a + b \log j \tag{S13}$$

Here, the overpotential η is the difference between the applied potential (E) and the equilibrium potential (E_{eq}), while the measured j reflects the reaction rate; a and b are the empirical Tafel constants. This Tafel relation is an essential basis for assessing reaction rate and investigating the potential-dependent kinetics of electrocatalytic processes.

The electrochemical reaction shown in eq (S14) is considered.

$$\mathrm{O} + \mathrm{e}^- \leftrightarrow \mathrm{R} \tag{S14}$$

The reaction rate follows the Butler-Volmer (B-V) equation under pure kinetic control (eq (S15)).[S16]

$$j = j_0 \left[\exp\left(-\frac{\beta F \eta}{RT}\right) - \exp\left(-\frac{(1-\beta) F \eta}{RT}\right) \right] \tag{S15}$$

Here, j_0 represents the current exchange density (the reaction rate when E equals E_{eq}), F is the Faraday constant (96485 C mol^{-1}), R is the gas constant (8.314 J mol^{-1} K^{-1}), T is the absolute temperature, and β is known as the symmetrical factor, typically ranging from 0 to 1. In the absence of precise information, it is commonly assumed that the overpotential affects the forward and reverse reactions equally, thus β is taken to be 0.5.[S5]

Only when η is sufficiently large, the current remains governed by the forward reaction, allowing the reverse reaction to be ignored. Hence, the B-V equation can be simplified to eq (S16).[S5-6]

$$j = j_0 \left[\exp\left(-\frac{\beta F \eta}{RT}\right) \right] \tag{S16}$$

Following logarithmic manipulation, eq (S16) can be reconfigured to yield the Tafel equation (eq (S17)).

$$\eta = \left(\frac{2.303RT}{\beta F}\right) \log j_0 + \left(-\frac{2.303RT}{\beta F}\right) \log j \tag{S17}$$

$$a = \left(\frac{2.303RT}{\beta F}\right) \log j_0 \tag{S18}$$

$$b = -\frac{2.303RT}{\beta F} \tag{S19}$$

The empirical Tafel constants a and b in eq (S13) can be derived as shown in eqs (S18 and (S19).

Therefore, the Tafel equation can be considered as a framework for analysing electron-transfer reactions. Tafel slope b obtained from the η-log j relationship reflects catalyst activity and provides key experimental evidence for identifying the RDS under kinetically controlled conditions.

Theoretical Tafel slopes are typically derived assuming that a single elementary step is RDS. For multi-electron processes, however, the observed Tafel slope value is strongly dependent on the identity of the RDS. In the case of eCO_2RR, the reaction proceeds via multiple electron-transfer and chemical transformation steps, each of which may control the overall rate. Moreover, various mechanistic pathways have been proposed, involving distinct adsorbed intermediates and corresponding to different RDSs, thereby yielding a range of Tafel slopes. The literature-reported mechanisms, their associated Tafel slopes, and rate expressions are summarized in **Table S4**,[S5-6,17] serving as diagnostic criteria for interpreting experimental observations and assigning the most probable RDS.

Assumption

This analysis assumes that the reaction proceeds under activation control, such that the current is not limited by the mass transfer of reactants to the electrode surface. Each elementary step was considered individually in the RDS, with all other steps treated as being in quasi-equilibrium. CO desorption was excluded as a possible RDS.[S5,18] A low surface coverage of any adsorbed intermediates was assumed, such that the fraction of unoccupied active sites (θ_M) approaches unity. A symmetrical factor β of 0.5 was applied. The analysis was further restricted to the low-overpotential region, where cation effects on active-site availability were considered negligible.

Dependence of Reaction Rate on Reactant Concentration

The derivation of explicit rate expressions quantifies how the reaction rate depends on the applied potential and the concentrations of relevant reactants or intermediates. Such formulations enable a direct connection between mechanistic models and measurable electrochemical parameters, thereby facilitating a more rigorous comparison between theoretical predictions and experimental observations. Importantly, the functional form of these dependencies provides diagnostic criteria for distinguishing between different RDS scenarios that may yield identical theoretical Tafel slopes.[S5,19] The rate expression can be formulated into a generalised form eq (S20).

$$j_{CO} = k\,[A]^z \qquad (S20)$$

where k is the rate constant and [A] represents the concentration of CO_2, proton donor, H^+, or intermediates; z is the apparent reaction order. Eq (S20) can be rearranged to a log-log version (eq (S21))

$$\log j_{CO} = \log k + z \log[A] \qquad (S21)$$

Therefore, the reaction order z can be determined from the slope of a log j_{CO} versus log [A] plot, which reflects the dependence of CO partial current density on the concentration of reactant A. This parameter quantifies the dependence of the CO partial current density on the availability of species A and thus indicates whether A is directly involved in the RDS. A reaction order close to zero suggests that the species does not participate in the RDS, whereas a positive or negative order implies a direct kinetic influence. When combined with Tafel slope analysis, reaction order measurements provide a complementary diagnostic framework, enabling mechanistic assignment to be cross-validated across both potential and concentration dependencies. This dual-parameter approach underpins the interpretation of subsequent experimental results and the assessment of the plausibility of different mechanistic pathways in bicarbonate electroreduction.

Although the theoretical framework described above was established based on CO_2 electroreduction, its application to bicarbonate (HCO_3^-) conversion is both appropriate and informative. In Ar-saturated $KHCO_3$ aqueous solutions, HCO_3^- serves as a practical carbon source in equilibrium with dissolved CO_2(aq), as demonstrated in this work. Therefore, the pre-equilibrium must be considered when performing theoretical calculations of rate expressions. In the system of bicarbonate conversion, the amount of DIC species, including HCO_3^-, CO_2(aq), and CO_3^{2-}, cannot be changed independently, and the bulk pH and the acid-base equilibrium rates among them determine their concentration near the electrode surface. Consistent with the assumptions underlying the theoretical Tafel derivation, the system must operate under kinetic control, with negligible mass-transport influence. The overall equilibrium between DIC species is extremely fast compared to the kinetic reactions,[S5] indicating that the concentration of CO_2(aq) near the electrode surface is not affected by eq (S22).

$$HCO_3^- + H^+ \rightleftharpoons CO_2(aq) + H_2O \qquad (S22)$$

Therefore, the [CO_2] in the rate expression can be substituted as shown in eq (S23).

$$[CO_2(aq)] = K_1[HCO_3^-][H^+] \quad (S23)$$

Here, K_1 equals the acid dissociation constant of carbonic acid, with pK_1 of 6.35 at 25 °C.[S10] This expression reveals that, under such conditions, the observed dependence of the rate on bicarbonate concentration (i.e., the apparent reaction order with respect to HCO_3^-) does not directly reflect its involvement in the elementary electrochemical step. Instead, the apparent reaction order arises from the acid-base equilibrium that governs the availability of CO_2(aq) at the electrode surface. Consequently, even if HCO_3^- is not directly involved in the RDS, a first-order dependence on $[HCO_3^-]$ may still be observed due to this pre-equilibrium. This has important implications for interpreting kinetic data and identifying the true rate-determining species.

References

(S1). Y.-J. Kong, A.-C. Zheng, Z. Wu, Q. Chen, X.-M. Hu, "Hollow Carbon Spheres with Isolated Ni Atoms on Both External and Internal Surfaces for Efficient CO_2 Electroreduction," *Materials Today Chemistry* 28, no. (2023): 101386, https://doi.org/10.1016/j.mtchem.2023.101386.

(S2). G. Katsoukis, H. Heida, M. Gutgesell, G. Mul, "Time-Resolved Infrared Spectroscopic Evidence for Interfacial pH-Dependent Kinetics of Formate Evolution on Cu Electrodes," *ACS Catalysis* 14, no. 18 (2024): 13867−13876, https://doi.org/10.1021/acscatal.4c03521.

(S3). A. R. Davis, B. G. Oliver, "A Vibrational-Spectroscopic Study of the Species Present in the CO_2−H_2O System," *Journal of Solution Chemistry* 1, no. 4 (1972): 329, https://doi.org/10.1007/BF00715991.

(S4). S. Mukhopadhyay, M. S. Naeem, G. Shiva Shanker, et al., "Local CO_2 Reservoir Layer Promotes Rapid and Selective Electrochemical CO_2 Reduction," *Nature Communications* 15, no. 1 (2024): 3397, https://doi.org/10.1038/s41467-024-47498-9.

(S5). A. Wuttig, Y. Yoon, J. Ryu, Y. Surendranath, "Bicarbonate Is Not a General Acid in Au-Catalyzed CO_2 Electroreduction," *Journal of the American Chemical Society* 139, no. 47 (2017): 17109, https://doi.org/10.1021/jacs.7b08345.

(S6). M. Dunwell, W. Luc, Y. Yan, F. Jiao, B. Xu, "Understanding Surface-Mediated Electrochemical Reactions: CO_2 Reduction and Beyond," *ACS Catalysis* 8, no. 9 (2018): 8121, https://doi.org/10.1021/acscatal.8b02181.

(S7). R. E. Zeebe, D. Wolf-Gladrow, *CO_2 in seawater: equilibrium, kinetics, isotopes*, Gulf Professional Publishing, **2001**.

(S8). J. Li, Y. Kuang, Y. Meng, et al., "Electroreduction of CO2 to Formate on a Copper-Based Electrocatalyst at High Pressures with High Energy Conversion Efficiency," *Journal of the American Chemical Society* 142, no. 16 (2020): 7276, https://doi.org/10.1021/jacs.0c00122.

(S9). J. N. Butler, *Carbon dioxide equilibria and their applications*, Routledge, **2019**.

(S10). S. K. Lower, "Carbonate equilibria in natural waters," *Simon Fraser University* 544, no. (1999): 1.

(S11). Y. Pocker, D. Bjorkquist, "Stopped-Flow Studies of Carbon Dioxide Hydration and Bicarbonate Dehydration in H_2O and D_2O. Acid-Base and Metal ion Catalysis," *Journal of the American Chemical Society* 99, no. 20 (1977): 6537−6543.

(S12). K. Adamczyk, M. Prémont-Schwarz, D. Pines, E. Pines, E. T. J. Nibbering, "Real-Time Observation of Carbonic Acid Formation in Aqueous Solution," *Science* 326, no. 5960 (2009): 1690−1694, https://doi.org/10.1126/science.1180060.

(S13). L. W. Diamond, N. N. Akinfiev, "Solubility of CO_2 in Water from− 1.5 to 100 °C and from 0.1 to 100 MPa: Evaluation of LiteratureData and Thermodynamic Modelling," *Fluid Phase Equilibria* 208, no. 1-2 (2003): 265−290.

(S14). W. L. Luyben, *Process Modeling,Simulation and Control for Chemical Engineers*, McGraw-Hill Higher Education, **1989**.

(S15). Y.-H. Fang, Z.-P. Liu, "Tafel Kinetics of Electrocatalytic Reactions: From Experiment to First-Principles," *ACS Catalysis* 4, no. 12 (2014): 4364, https://doi.org/10.1021/cs501312v.

(S16). A. J. Bard, L. R. Faulkner, H. S. White, *Electrochemical Methods: Fundamentals and Applications*, John Wiley & Sons, **2022**.

(S17). M. D. Hossain, Y. Huang, T. H. Yu, W. A. Goddard, III, Z. Luo, "Reaction Mechanism and Kinetics for CO_2 Reduction on Nickel Single Atom Catalysts from Quantum Mechanics," *Nature Communications* 11, no. 1 (2020): 2256, https://doi.org/10.1038/s41467-020-16119-6.

(S18). A. Wuttig, M. Yaguchi, K. Motobayashi, M. Osawa, Y. Surendranath, "Inhibited Proton Transfer Enhances Au-Catalyzed CO_2-to-Fuels Selectivity," *Proceedings of the National Academy of Sciences of the United States of America* 113, no. 32 (2016): E4585, https://doi.org/doi:10.1073/pnas.1602984113.

(S19). T. Li, C. Yang, J.-L. Luo, G. Zheng, "Electrolyte Driven Highly Selective CO2 Electroreduction at Low Overpotentials," *ACS Catalysis* 9, no. 11 (2019): 10440, https://doi.org/10.1021/acscatal.9b02443.
